# *Thaldh3*-Dependent GABA Metabolism Modulates Response of *Trichoderma* to Fusaric Acid-Induced Oxidative Stress

**DOI:** 10.3390/jof11070542

**Published:** 2025-07-21

**Authors:** Linhua Cao, Xiaoteng Shi, Tuo Li, Yang Liu, Tuokai Wang, Bozheng Lin, Dongyang Liu, Qirong Shen

**Affiliations:** 1 Key Lab of Organic-Based Fertilizers of China and Jiangsu Provincial Key Lab for Solid Organic Waste Utilization, Nanjing Agricultural University, Nanjing 210095, China; 2 College of Resources & Environmental Science, Nanjing Agricultural University, Nanjing 210095, China

**Keywords:** *Trichoderma harzianum* NJAU4742, *Thaldh3*, GABA, fusaric acid, plant disease

## Abstract

Fusaric acid (FSA) is a mycotoxin produced by pathogenic *Fusarium* species that inhibits the growth of various beneficial microbes. In this study, we investigated the molecular mechanisms by which *Trichoderma harzianum* NJAU4742 (*Th*), a beneficial fungus, responds to FSA-induced stress. Here, by combining a transcriptome analysis, a gene knockout, and physiological data measurements, our study investigated the molecular mechanisms underlying the response of *Trichoderma harzianum* NJAU4742 (*Th*) to FSA stress. The results showed that FSA can induce severe oxidative stress in *Th*, and an aldehyde dehydrogenase (*Thaldh3*) in *Th* plays a critical role in alleviating FSA stress. Deleting *Thaldh3* significantly decreased the γ-aminobutyrate (GABA) content, causing more severe oxidative damage in *Th*. Furthermore, we also provide evidence demonstrating that *Thaldh3* alleviates FSA stress by enhancing the activities of key enzymes involved in the tricarboxylic acid cycle and ATP content. A pot experiment showed that an enhanced tolerance to FSA increased the *Th* biomass, strengthening its antagonistic capacity against pathogens and reducing the disease index in tomatoes. In conclusion, these observations provide new insight into the role of beneficial microbes in promoting plant health.

## 1. Introduction

*Fusarium* species are the most significant plant pathogens among the world, capable of infecting a wide range of ~100 host plant species, including maize, tomatoes, wheat, cotton, rice, bananas, and eggplants. They cause severe damage in terms of global food security [1,2,3]. *Fusarium* species possess the genetic capacity to produce hundreds of structurally diverse secondary metabolites, including various mycotoxins such as trichothecenes, zearalenone, fumonisins, enniatins, and fusaric acid (FSA) [1,4]. These toxins are widely regarded as critical factors contributing to plant disease development [5]. Moreover, it has been reported that *Fusarium* infections often lead to substantial toxin accumulation in crops, which not only significantly reduces both the quality and yield of agricultural products [6], but also poses a severe threat to humans and livestock [7]. FSA is a non-specific phytotoxin produced by several *Fusarium* species that exhibits strong cytotoxicity to both plant and animal cells [8]. The phytotoxic effects of FSA on plants include damaging host cell membranes [9], reducing mitochondrial respiratory activity [10], and inhibiting ATP synthesis [9]. In animals, FSA toxicity typically manifests as vomiting and hypotension [11]. Therefore, it is imperative to develop effective strategies to manage *Fusarium* and food contamination by its mycotoxins.

Currently, the prevailing strategy for controlling *Fusarium* relies on the utilization of synthetic agrochemicals [12]. This approach not only intensifies the emergence of fungicide resistance, but also results in detrimental effects on ecosystems. The application of microbial biological agents (BCAs) is regarded as an environmentally friendly measure to manage Fusarium wilt disease [13]. Various BCAs have been reported to control various pathogens through mechanisms such as inhibiting pathogen growth [14], suppressing mycotoxin synthesis [15], and activating plant defense responses [16]. However, during the antagonistic interactions between BCAs and *Fusarium*, the production of inhibitory compounds capable of halting or impeding the growth of competing microorganisms provides an ecological advantage to the producing microbes [17]. This specific form of intense competition is referred to as “interference competition” [18]. Mycotoxins secreted by *Fusarium*, especially enniatins, beauvericin, and FSA, exhibit antimicrobial activity against a variety of BCAs. For example, enniatins were found to promote the inhibition of *B. bassiana* and *T. harzianum* [19]. Beauvericin had a strong antibacterial activity against plant growth bacteria and Gram-negative bacteria [20]. Therefore, enhancing the resistance of BCAs to mycotoxins is a critical factor for improving their biocontrol efficacy against pathogens.

*Trichoderma* is one of the most widely used BCAs and biofertilizers in agricultural ecosystems worldwide [21]. It can colonize in the plant rhizosphere, thereby promoting plant growth and defending against various plant diseases [16,21]. *Trichoderma harzianum* NJAU4742 (*Th*), initially isolated from mature compost, has been reported to inhibit pathogen growth through mycoparasitism [22] and the secretion of chemical compounds [23]. However, these studies mainly focused on the direct interaction between *Th* and pathogens; the details of the mechanism by which *Th* tolerates FSA secreted by *Fusarium* pathogens remain largely unclear. A previous study reported that *Trichoderma* has the ability to tolerate various inorganic and organic environmental pollutants [24]. In this study, we proved that *Th* was able to grow under a high FSA concentration. The transcriptomics showed that an aldehyde dehydrogenase (*aldh3*) gene responsible for γ-aminobutyrate (GABA) synthesis played a crucial role in defending against FSA. GABA is a widely distributed four-carbon non-protein amino acid that is associated with various abiotic and biotic stresses [25]. For instance, the accumulation of GABA directly interacts with aluminum-activated malate transporters and outward-rectifying K^+^ channels within guard cells, thereby enhancing drought and hypoxia tolerance in plants [25]. The application of GABA is reported to enhance ion transport, cell wall remodeling, antioxidation, growth and reproduction, stress resistance, and amino acid synthesis in yeast [26]. Therefore, the objective of this study was to elucidate the molecular mechanisms by which *Trichoderma harzianum* tolerates fusaric acid toxicity, with a specific focus on the role of the *Thaldh3*-mediated GABA pathway. Additionally, we aimed to clarify how this specific mechanism contributes to the antagonistic interaction between *Th* and *Fusarium* pathogens, thereby deepening our understanding of microbial interactions and biocontrol efficacy in the rhizosphere.

## 2. Materials and Methods

### 2.1. Strains and Culture Conditions

*Trichoderma harzianum* NJAU4742 (*Th*) was isolated from mature compost and stored at the Jiangsu Provincial Key Lab for Organic Solid Waste Utilization. The genome sequence (NCBI accession: LVVK00000000.1) has been previously published [23]. *Th* was cultured on potato dextrose agar (PDA; Difco, Franklin Lakes, NJ, USA) at 28 °C, and spores were harvested by washing 5-day-old cultures with sterile water, filtered through four layers of sterile gauze, and quantified using a hemocytometer (Marienfeld, Lauda-Königshofen Germany).

*Fusarium oxysporum* f. sp. *lycopersici* strain Fol4287 (*Fol*) (stored at the Jiangsu Provincial Key Lab for Organic Solid Waste Utilization) was cultured on PDA under identical conditions, with the conidia prepared as described above.

### 2.2. Inhibitory Effects of FSA on Th

To determine the inhibitory effects of FSA (Sigma, Darmstadt, Germany) on *Th*, fresh *Th* spores were inoculated onto minimal media (MM) containing glucose (1%, *w*/*v*), KH_2_PO_4_ (1.5%, *w*/*v*), (NH_4_)_2_SO_4_ (0.5%, *w*/*v*), and MgSO_4_ (0.06%, *w*/*v*), and the concentration of FSA was adjusted using a diluted FSA solution (100 mg mL^−1^). Three days after inoculation, the inhibitory rate of FSA on *Th* was calculated. Each treatment included three independent biological replicates.

### 2.3. RNA Sequencing and Transcriptome Analysis

Spores of *Th*-WT (1 × 10^7^ spores mL^−1^) were inoculated with or without FSA (0.3 mg mL^−1^) for three days. The mycelia of *Th*-WT in two treatments were collected and flash-frozen in liquid nitrogen for a further transcriptional analysis. For each treatment, three independent biological replicates were prepared for RNA extraction and transcriptome sequencing. The total RNA was extracted using the mirVana miRNA Isolation Kit (Ambion, Austin, TX, USA), and the integrity was verified by 1% agarose gel electrophoresis. Qualified RNA samples were sequenced on an Illumina sequencing platform (HiSeqTM 2500, Illumina, Inc., San Diego, CA, USA). High-quality clean reads were filtered using Cutadapt (version 1.9). Raw reads were quality-filtered with Cutadapt (v1.9) and aligned to the *T. harzianum* NJAU4742 genome using HISAT2 (version 2.2.1.0). Differentially expressed genes (DEGs) were identified using the DESeq (2012) R package (version 1.18.0), and the significance thresholds were set at a *p*-value < 0.05 and a fold change ≥ 1.50 or ≤0.67. Based on the results of DEGs, volcano plots were created and KEGG (Kyoto Encyclopedia of Genes and Genomes) enrichment analyses were conducted to explore the functional roles of the selected genes. All the analyses and visualizations were carried out using R. The data were deposited to the NCBI under the accession number PRJNA1189886.

### 2.4. Extraction of T. harzianum NJAU4742 Genomic DNA and Construction of Homologous Fragments

Genomic DNA of *T. harzianum* NJAU4742 was extracted using the CTAB method. The fungal mycelia were first frozen in liquid nitrogen and then ground thoroughly using a mortar and pestle. Approximately 200 mg of the resulting powder was transferred to a tube containing 500 μL of CTAB extraction buffer. The mixture was incubated at 65 °C for 30 min, with mixing every 10 min. After incubation, 200 μL of chloroform was added, and the solution was gently mixed at room temperature for 2 min. The mixture was then centrifuged at 12,000× *g* for 5 min, and the supernatant was carefully collected. An equal volume of isopropanol was added to the supernatant, followed by incubation at −20 °C for at least 30 min. Afterward, the solution was centrifuged at 12,000× *g* for 5 min, and the supernatant was discarded. The resulting pellet was washed with 700 μL of 75% ethanol, vortexed briefly, and centrifuged again at 12,000× *g* for 5 min. The supernatant was discarded, and the tube containing the DNA pellet was air-dried in a biosafety cabinet (UV light turned off). Once dry, the pellet was resuspended in 100 μL of deionized water by vortexing, and the DNA concentration was determined using a UV spectrophotometer.

The construction of homologous fragments was generated using overlapping PCR. The 1.2 kb upstream and downstream flanking regions of target genes were amplified using Phanta Flash Master Mix (Vazyme P520-01, Vazyme, Nanjing, China). The fusion construct was organized in the following order: upstream–hygromycin B–downstream. Overlapping PCR was performed using the CloneAmp HiFi PCR Premix (TaKaRa, San Jose, CA, USA, Cat. No. 639298). The primers used for the overlapping amplification are listed in Supplementary Table S1.

### 2.5. Generation of Targeted Gene Mutants in T. harzianum NJAU4742

A schematic of the gene knockout strategy is presented in Supplementary Figure S1. *Th*-WT spores (1 × 10^8^ spores mL^−1^) were inoculated and evenly spread onto solid PDA medium covered with a sterile cellophane sheet. After approximately 16 h of incubation, the germinated spores were lysed using a lysis solution composed of 0.75% (*w*/*v*) lysing enzymes (Sigma, Lot# SLBJ0553V) and Solution A (6 M sorbitol, 0.5 M KH_2_PO_4_, and a pH adjusted to 5.6 with 1 M KOH). The lysis mixture was incubated at 28 °C with shaking at 100 rpm for 120 min. Following lysis, the spore layer was gently dislodged using pipette tips and filtered through four layers of sterile gauze on ice. The resulting filtrate was then centrifuged at 2000 rpm for 10 min at 4 °C. The supernatant was retained to a final volume of 3 mL, and the pellet was gently resuspended. The suspension was centrifuged again under the same conditions, and the supernatant was discarded. The remaining pellet was resuspended in Solution B (10 M sorbitol, 0.5 M CaCl_2_·2H_2_O, and 1 mL of 1 M Tris–HCl, with a pH of 7.5) to prepare the protoplast suspension.

Protoplast transformation was carried out by mixing the protoplast solution with functional DNA fragments and a PEG solution (25% PEG6000, 0.05 M CaCl_2_·2H_2_O, and 1 mL of 1 M Tris–HCl, with a pH of 7.5) in a volume ratio of 77% protoplast solution, 19% PEG solution, and 4% DNA fragments. This mixture was incubated on ice for 20 min, followed by the addition of 4 mL of the PEG solution and another 5 min incubation on ice. Subsequently, 6 mL of Solution B was added. The transformation mixture was then spread evenly onto sucrose–PDA plates (containing 1 M sucrose). After approximately 16 h, PDA medium supplemented with 0.2 mg mL^−1^ of hygromycin B was poured over the surface. After 36 h, emerging transformants were transferred to a selective PDA medium. Homologous recombinations were confirmed via PCR to eliminate non-specific insertions. Verified transformants were purified by single-spore isolation on a selective medium, ensuring the presence of the target gene and clonal purity.

### 2.6. Assay of Mycelium Inhibition Growth Rate and Intracellular Metabolites

To assess the sensitivity of various strains to fusaric acid (FSA), 1 μL of spore suspension (1 × 10^7^ spores mL^−1^) from *T. harzianum* and its mutants was inoculated onto MM medium supplemented with FSA, achieving a final concentration of 0.3 mg mL^−1^. After six days, the diameters of the colonies were measured. To reveal specific growth-suppression patterns across individual gene knockout strains under FSA exposure, the specific index was measured as follows: specific index = (mean diameter of *Th*-WT in FSA treatment − mean diameter of *Th*-mutant in FSA treatment)/(mean diameter of *Th*-WT in control treatment − mean diameter of *Th*-mutant in control treatment). Each treatment included three independent biological replicates.

Similarly, to evaluate the contributions of GABA and β-alanine in alleviating FSA stress, the mycelial diameter was measured after four days of culture on MM medium supplemented with FSA and additional GABA (100 μg mL^−1^) or β-alanine (100 μg mL^−1^), respectively. The entire process included a control group in which FSA was not incorporated. Simultaneously, fresh mycelia were harvested from MM plates covered with cellophane. The mycelia were then homogenized in ice-cold extraction buffer (Solarbio BC0310 kit, Solarbio, Guntur, India), followed by centrifugation at 12,000× *g* for 10 min. The resulting supernatants were analyzed for metabolites using commercial kits: superoxide dismutase (SOD) (Solarbio BC0175), reduced glutathione (GSH) (Solarbio BC1175), oxidized glutathione (GSSG) (Solarbio BC1180), citrate synthase (CS) (Solarbio BC1060), isocitrate dehydrogenase (ICDHm) (Aladdin I486212-1kit, Aladdin Scientific, Riverside, CA, USA), α-ketoglutarate dehydrogenase (α-KGDH) (CheKine KTB1240, Abbkine, Atlanta, GA, USA), γ-aminobutyric acid (GABA) (Elabscience E-BC-K852-M, Elabscience, Wuhan, China), glutamate decarboxylase (GAD) (geruisi G1102F, Geruisi, Hangzhou, China), γ-aminobutyric acid transaminase (GABA-T) (geruisi G1103F), and adenosine triphosphate (ATP) (CheKine KTB1019). Each treatment included three independent biological replicates, and each biological replicate consisted of three technical replicates.

### 2.7. Pot Experiments

Soil from a tomato-cultivated field (Baima Experimental Base, Nanjing, China) was air-dried, sieved (2 mm), and split into non-sterile and γ-irradiated (Co_60_, 25 kGy) batches. Surface-sterilized *Solanum lycopersicum* cv. Micro-Tom seeds (70% ethanol, 3 min; 5% NaOCl, 3 min) were germinated on moist filter paper for 2 days and grown in seedling trays for 7 days. Polypropylene pots (200 g soil) received four treatments, as follows:

1. T1 (CK): Soil inoculated with *Fol* only, serving as the control.
2. T2: Soil co-inoculated with *Fol* and *Th*-WT strains.
3. T3: Soil co-inoculated with *Fol* and the Δ*Thaldh3* strains.
4. T4: Soil co-inoculated with *Fol* and Δ*Thaldh3*, with exogenous GABA added at 100 μg g^−1^ soil.

Each treatment included three replicates with eight plants in each replicate. Spores of *Th*-WT or its mutant, Δ*Thaldh3* (1 × 10^6^ spores g^−1^ soil), were applied 3 days post-transplantation. *Fol* (1 × 10^6^ spores g^−1^ soil) was inoculated 7 days later. The plants were maintained in a growth chamber (16 h light/30 °C, 8 h dark/28 °C, 75% humidity). The disease severity index (DI) of the tomato plants was evaluated according to the scoring scale described by Ji et al. [27]. In brief, Scale 0 represented healthy plants without any signs of wilting or yellowing. Scale 1 indicated the wilting or abscission of cotyledons. Scale 2 was assigned when 30–50% of the true leaves were wilted or dropped. Scale 3 corresponded to 50–80% wilting or the loss of true leaves, and Scale 4 denoted complete leaf abscission or the death of the entire plant. The disease severity index of the tomato plants was assessed using the following formula:

$$DI=\left[\frac{\sum_{\;i=1}^{\;k}\left(sᵢ\times nᵢ\right)}{N\times Sₘₐₓ}\right]\times 100\%$$

where

*s_i_* = severity score of the *i*-th category;

*n_i_* = number of plants in the *i*-th severity category;

*N* = total number of plants assessed;

*S*_max_ = maximum severity score possible;

*k* = number of severity categories.

### 2.8. Soil Sampling, DNA Extraction, and Real-Time Quantitative PCR Analysis

The rhizosphere soils were collected as previously described. DNA was extracted from 0.5 g of soil from each sample using the PowerSoil DNA Isolation Kit (Mobio Laboratories, Carlsbad, CA, USA) according to the manufacturer’s instructions. The DNA concentration and quality were assessed with a NanoDrop 2000 spectrophotometer (Thermo Scientific, Waltham, MA, USA). The abundances of *Fol* and *Th* were measured on an ABI 7500 real-time PCR system (Applied Biosystems, Waltham, MA, USA). The primers used in this study are shown in Supplementary Table S1.

### 2.9. Statistical Analyses

All the statistical analyses were conducted using IBM SPSS version 22 (IBM Corporation, New York, NY, USA) and the R software (version 3.5.0). The significance of the differences among multiple groups, excluding the sequencing data, was assessed using a one-way analysis of variance (ANOVA) followed by Duncan’s multiple range tests. For comparisons between two groups, a two-sided Student’s *t*-test was employed. The following thresholds were applied throughout the study: *p* < 0.05 (*) and n.s. indicating no statistical significance.

## 3. Results

### 3.1. The Transcriptome Profiling of Th Exposed to FSA

**Figure 1:**
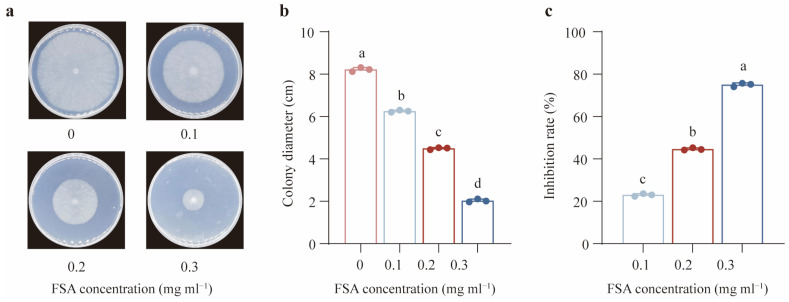
The influence of fusaric acid (FSA) on the growth of *T. harzianum* NJAU4742. (**a**) An image of *T. harzianum* NJAU4742 grown under different FSA concentrations. (**b**) The colony diameter of *T. harzianum* NJAU4742 under different FSA concentrations. (**c**) The inhibition rate of *T. harzianum* NJAU4742 under different FSA concentrations. The strains were inoculated on MM medium supplemented with 0, 0.1, 0.2, or 0.3 mg mL^−1^ of FSA for 72 h. The data represent the means ± s.d.; *n* = 3 (**b**,**c**). Different letters above the error bars indicate significant groups (*p* < 0.05), one-way ANOVA, Duncan’s multiple range tests, (**b**,**c**).

To investigate the influence of FSA on the growth of *Th*, the *Th* was treated with different FSA concentrations (0.1 to 0.3 mg·mL^−1^). As shown in Figure 1a,b, FSA substantially inhibited the mycelial growth of *Th* in a dose-dependent manner. The highest inhibition rate was 75%, which occurred when *Th* was exposed to 0.3 mg mL^−1^ of FSA (Figure 1c). To gain a more comprehensive understanding of the mechanisms by which *Th* tolerates the extreme toxicity of FSA, we determined 0.3 mg mL^−1^ as the optimal concentration for subsequent experiments. Subsequently, the transcriptome profiling was performed on *Th* exposed to this concentration of FSA.

**Figure 2:**
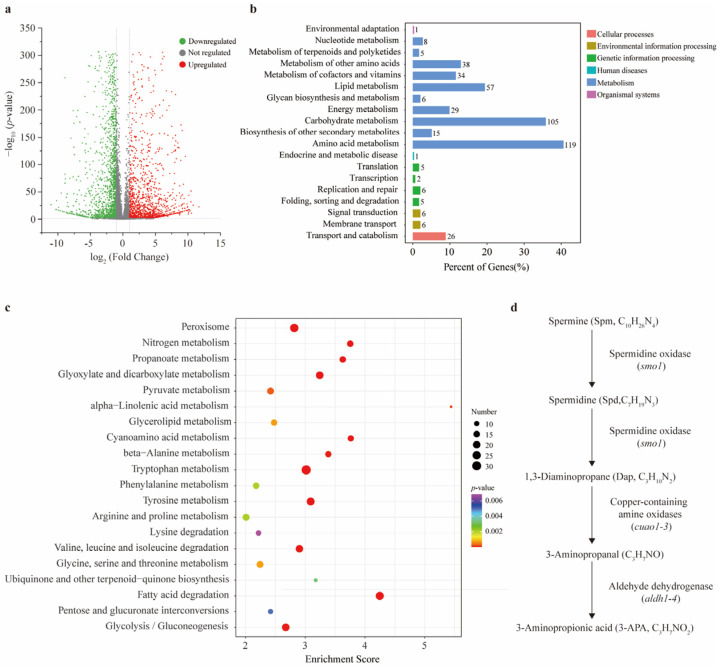
The transcriptome analysis of *T. harzianum* NJAU4742 under fusaric acid (FSA) stress. (**a**) Volcano plot of all identified genes between CK and FSA treatments. Grey circles indicate no regulated expressed genes, green circles denote significantly downregulated genes, and red circles indicate significantly upregulated genes. The horizontal axis represents the log_2_ fold change (log_2_FoldChange), and the vertical axis represents the negative base −10 logarithm of the *p*-value (−log_10_ *p*-value). Dashed lines indicate the significance thresholds corresponding to a *p*-value < 0.05 and an absolute fold change of ≥1.50 (upregulation) or ≤0.67 (downregulation). (**b**) The primary functional categories of the significant upregulated genes according to the Kyoto Encyclopedia of Genes and Genomes (KEGG) analysis. (**c**) The bubble map of the top 20 upregulated subcategories belonging to substance metabolism. The size of the circles represents the count of enriched genes. (**d**) A specific segment of substance metabolism belonging to beta-alanine metabolism was consistently upregulated.

According to the statistical assessment, more than 93% of the sample data were valid, and the sequencing quality (Q30) exceeded 95%, demonstrating that the dataset satisfied the required quality criteria (Supplementary Table S2). The principal coordinate analysis (PCoA) results revealed a significant transcriptional difference between CK and the FSA treatment, and this finding was further validated by the correlation coefficient matrix (Supplementary Figures S2 and S3). To further illustrate the differences between the two treatments, a volcano plot analysis was performed. The results showed that a total of 3443 unigenes exhibited significant expression changes following the FSA treatment, with 2063 unigenes upregulated and 1380 unigenes downregulated (Figure 2a). A Kyoto Encyclopedia of Genes and Genomes (KEGG) analysis classified the unigenes into six primary functional categories, including cellular processes, environmental information processing, genetic information processing, human diseases, metabolism, and organismal systems. The most significantly upregulated subcategories are presented in Figure 2b, and 10 of them were associated with metabolism. Meanwhile, we found that the most upregulated gene pathways were also linked to substance metabolism (Figure 2c). Based on these observations, we thus focused on the gene expression related to metabolism for further analysis. Notably, we observed that, within the KEGG pathways associated with beta-alanine metabolism, a specific segment of the substance metabolism was consistently upregulated (Supplementary Figure S4). All eight genes involved in this metabolism were significantly upregulated in response to the FSA treatment based on our transcriptomic analysis. The fold change in expression ranged from 2.98 to 8.88, indicating the robust transcriptional activation of this metabolic pathway under FSA-induced oxidative stress. This metabolic cascade encompasses the sequential changes from spermine to spermidine, followed by the conversion of spermidine into 1, 3-diaminopropane, then 1, 3-diaminopropane into 3-aminopropanal, and finally, 3-aminopropanal into 3aminopropionic acid (Figure 2d). Among these metabolites, spermidine and 3-aminopropionic acid (3-APA) are both recognized as well-known antioxidants under stress conditions. We thus inferred that *Th* may alleviate the inhibitory effects of FSA by activating the metabolic pathway of these key compounds.

### 3.2. FSA Induced Severe Intracellular Oxidative Stress in Th

**Figure 3:**
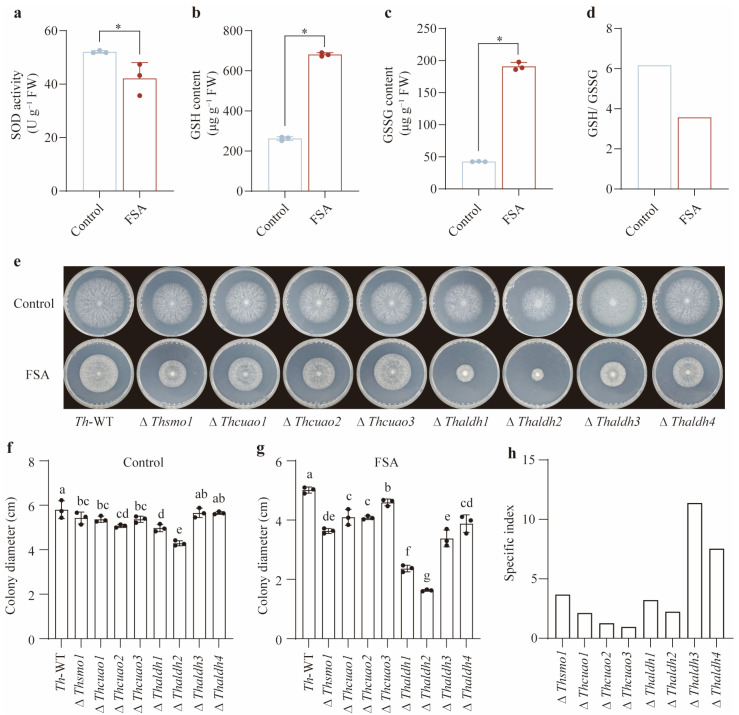
*Thaldh3* was responsible for mitigating FSA-induced oxidative stress. (**a**–**d**) Comparison of intracellular oxidative stress in *Trichoderma harzianum* NJAU4742 (*Th*) under the control and FSA (0.3 mg mL^−1^) treatments. The intracellular SOD activity (**a**), GSH content (**b**), GSSG content (**c**), and GSH /GSSG ratio (**d**) were measured. (**e**) The image of *T. harzianum* NJAU4742 (*Th*-WT) and its eight mutants grown under the control and FSA treatments. The strains were inoculated on MM medium for 72 h in the control treatment and on MM medium supplemented with 0.3 mg mL^−1^ FSA for 144 h in the FSA treatment. (**f**,**g**) The colony diameter of each strain in the control (**f**) and FSA (**g**) treatments. (**h**) The specific index to reveal specific growth-suppression patterns across individual gene knockout strains under FSA exposure. Each value was calculated using the mean colony diameters from three biological replicates. Since the index was derived from averaged values, statistical significance was not applied to this calculation. The data represent the means ± s.d.; *n* = 3 (**a**–**c**,**f**,**g**). Significance was determined by using a two-sided Student’s *t*-test (* *p* < 0.05). Different letters above error bars indicate a significant group (*p* < 0.05, one-way ANOVA, Duncan’s multiple range tests) (**f**,**g**).

To validate the above hypothesis, we examined whether FSA induces intracellular oxidative stress in *Th*. SOD is commonly regarded as the first line of defense against oxidative stress. Contrary to our expectations, we found that the application of FSA significantly decreased the intracellular SOD activity (*p* < 0.05, Figure 3a). This may be attributed to the fact that a high concentration of FSA causes excessive oxidative stress, which in turn inhibits SOD activity. Comparable findings have been documented in plants exposed to heavy metal toxicity and salt stress. Reduced glutathione (GSH), a ubiquitous protective antioxidant present in both intracellular and extracellular environments, serves as a critical cellular defense component. The GSH/ GSSG (oxidized glutathione) ratio is widely considered as a reliable biomarker for oxidative stress, where diminished ratios are correlated with heightened oxidative perturbation. We then selected the GSH/ GSSG ratio as the key indicator for quantifying the oxidative stress dynamics in *Th*. Our results demonstrated that FSA induced the marked upregulation of both the GSH and GSSG levels (*p* < 0.05), while concurrently eliciting a significant decrease in the GSH/ GSSG ratio compared to the control treatment (Figure 3b–d), unequivocally demonstrating FSA-triggered intracellular oxidative stress in *Th*.

### 3.3. Thaldh3 Played a Critical Role in the Response to FSA Stress

To functionally characterize the roles of various genes involved in the biosynthetic pathway from arginine to 3-aminopropionic acid in mitigating FSA-induced oxidative stress, we systematically constructed knockout mutants of all eight genes encoding this metabolic cascade. Except for the ∆*Thaldh3* and ∆*Thaldh4* mutants, all the other mutants exhibited varying degrees of growth impairment compared to *Th*-WT under the control treatment. Among them, ∆*Thaldh2* displayed the most severe growth impairment, followed by ∆*Thaldh1* and ∆*Thcuao2*, while no significant differences were observed among ∆*Thsmo1*, ∆*Thcuao1*, and ∆*Thcuao3* (Figure 3e,f). For the FSA treatment, similar growth impairments were observed among all the *Th* mutants compared to *Th*-WT. ∆*Thaldh2* and ∆*Thaldh1* exhibited the most severe growth impairment, followed by ∆*Thaldh3* and ∆*Thsmo1*, with no significant differences among ∆*Thcuao1*, ∆*Thcuao2*, and ∆*Thaldh4* (Figure 3e,g). Notably, ∆*Thcuao3* showed the slightest growth impairment. Due to a growth impairment caused by specific gene deletions in the control treatment, we employed a specific index (D_FSA-wt_ − D_FSA-mutant_)/(D_Control-wt_ − D_Control-mutant_) to reveal specific growth-suppression patterns across individual gene knockout strains under FSA exposure. Notably, we found that ∆*Thaldh3* showed the highest specific index among these mutants under the FSA treatment (Figure 3h), illustrating that the *Thaldh3* gene plays a critical role in FSA resistance. Based on these results, we selected ∆*Thaldh3* as the potential key gene for subsequent mechanistic investigations of oxidative stress mitigation.

### 3.4. GABA Was the Key Substance to Mitigate FSA-Triggered Oxidative Stress

**Figure 4:**
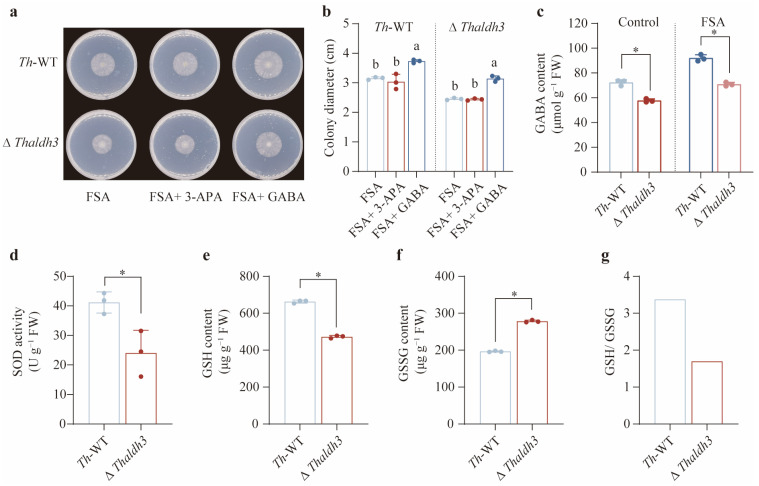
*Thaldh3* alleviated FSA-induced oxidative stress through regulating GABA content. (**a**) An image of *T. harzianum* NJAU4742 (*Th*–WT) and Δ*Thaldh3* grown under FSA, FSA + 3-APA, and FSA + GABA treatments. The strains were inoculated for 96 h in each treatment. (**b**) The colony diameter of *Th*-WT and Δ*Thaldh3* under the FSA, FSA+3-APA, and FSA+GABA treatments. (**c**) The intracellular GABA content in *Th*-WT and Δ*Thaldh3* under the control and FSA treatments. (**d**–**g**) A comparison of intracellular oxidative stress in *Th-WT* and Δ*Thaldh3* under FSA (0.3 mg mL^−1^) treatments. The intracellular SOD activity (**d**), GSH content (**e**), GSSG content (**f**), and GSH/ GSSG ratio (**g**) were measured. The data represent the means ± s.d.; *n* = 3 (**b**–**f**). Different letters above error bars indicate a significant group (*p* < 0.05, one-way ANOVA, Duncan’s multiple range tests) (**b**,**c**). Significance was determined by using a two-sided Student’s *t*-test (* *p* < 0.05) (**d**–**f**).

Given that *Thaldh3* encodes the enzyme responsible for converting 3-aminopropanal into 3-APA, the observed growth impairment in Δ*Thaldh3* under the FSA treatment is likely attributable to its reduction in the 3-APA content. Contrary to our expectation, an exogenous application of 3-APA failed to rescue the growth of *Th*-WT and Δ*Thaldh3* in the FSA treatment (Figure 4a,b). We then speculated that 3-APA is not the key substance used by *Th* to resist the oxidative stress caused by FSA. Previous studies have demonstrated the broad substrate specificity of the ALDH-family enzymes. Our results found that Δ*Thaldh3* significantly reduced the content of GABA (*p* < 0.05), a homolog of 3-APA, compared with *Th*-WT in both the CK and FSA treatments (Figure 4c). Furthermore, additive GABA markedly enhanced the *Th*-WT biomass and rescued Δ*Thaldh3* growth for *Th*-WT under FSA stress (*p* < 0.05, Figure 4a,b). These results suggest that *Thaldh3* mitigates FSA-induced stress by mediating the biosynthesis of GABA.

To confirm whether the *aldh3* knockout alleviated the intracellular oxidative stress caused by FSA, we analyzed the glutathione redox status by quantifying the SOD activity and the GSH/ GSSG ratios between Δ*Thaldh3* and *Th*-WT under FSA stress. Our results indicated that the SOD activity in Δ*Thaldh3* was significantly lower than in *Th*-WT (*p* < 0.05, Figure 4d). The Δ*Thaldh3* strain showed a significantly lower GSH content and higher GSSG accumulation compared to *Th*-WT under FSA stress (*p* < 0.05), resulting in a significantly reduced GSH/ GSSG ratio (Figure 4e–g). These results imply that GABA is essential for *Th* to counteract FSA-induced oxidative stress.

### 3.5. Thaldh3 Alleviated FSA Stress by Upregulating the Tricarboxylic Acid Cycle Rate

**Figure 5:**
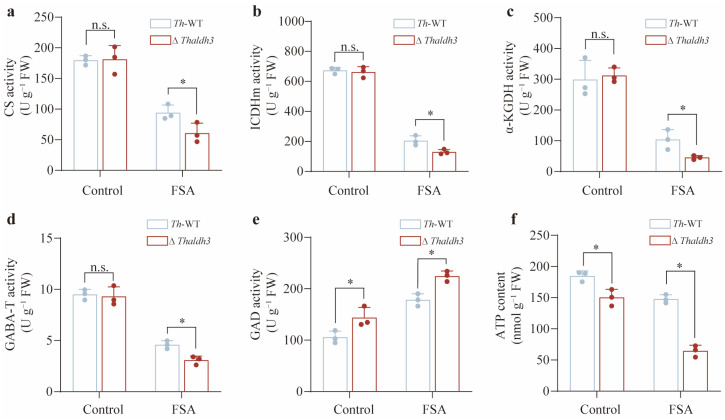
*Thaldh3* influenced the TCA cycle rates to alleviate FSA stress. The CS activity (**a**), ICDHm activity (**b**), α-KGDH activity (**c**), GABA-T activity (**d**), GAD activity (**e**), and ATP content (**f**) were measured in *Trichoderma harzianum* NJAU4742 (*Th*–WT) and Δ*Thaldh3* under the control and FSA treatments. The data represent the means ± s.d.; *n* = 3 (**a**–**f**). Significance was determined by using a two-sided Student’s *t*-test (* *p* < 0.05; n.s.: not significant) (**a**–**f**).

GABA is typically accumulated in the cytoplasm due to the oxidative stress. An increasing body of evidence suggests that GABA can be transported to the mitochondria, and then participate in the tricarboxylic acid (TCA) cycle. The next objective was to investigate the potential role of *Thaldh3* in influencing the TCA cycle under FSA-induced oxidative stress. We selected citrate synthase (CS), isocitrate dehydrogenase (ICDHm), and α-ketoglutarate dehydrogenase (α-KGDH) to characterize the rate of the TCA cycle. CS was the initial rate-limiting enzyme of the TCA cycle, which catalyzed the condensation of acetyl-CoA and oxaloacetate to form citrate. ICDHm and α-KGDH played critical roles in generating intermediate metabolites during the TCA cycle, which catalyzed the conversion of isocitrate into α-ketoglutarate (α-KG) and α-ketoglutarate into succinyl-CoA, respectively. Our results showed that there was no significant difference among these enzyme activities between *Th*-WT and Δ*Thaldh3* for the control treatment (Figure 5a–c). However, these enzyme activities in Δ*Thaldh3* were significantly lower than in *Th*-WT for the FSA treatment (*p* < 0.05, Figure 5a–c). These results indicate that *Thaldh3* enhanced the activities of key TCA-cycle-related enzymes by promoting GABA synthesis under FSA stress.

To further investigate the potential connection between *Thaldh3* and the TCA cycle, we also measured the enzyme activities of GABA transaminase (GABA-T) and glutamic acid decarboxylase (GAD). GABA-T catalyzes the transamination of GABA to succinic semialdehyde, which is further metabolized to succinate and enters the TCA cycle. GAD is responsible for converting glutamate to GABA, representing another pathway in GABA synthesis. Our study demonstrated that the GABA-T activity was not altered by the control treatment, but its activity in Δ*Thaldh3* was significantly lower than *Th*-WT for the FSA treatment (*p* < 0.05, Figure 5d). This might be attributable to the critical role of GABA under FSA stress. The knockout of *aldh3* significantly reduced the GABA content, which in turn led to a decrease in GABA-T activity. As for the GAD activity, it was significantly upregulated in Δ*Thaldh3* under both the CK and FSA treatments (*p* < 0.05, Figure 5e). As an alternative pathway for GABA synthesis, the significant upregulation of GAD activity was considered a compensation strategy in microbes. Given that the TCA cycle rate directly influences intracellular ATP production, we further measured the ATP content in *Th*. As anticipated, the ATP content showed a moderate decline between *Th*-WT and Δ*Thaldh3* in the control treatments. However, the difference between the two strains became much more pronounced for the FSA treatment (Figure 5f). In summary, our results revealed a *Thaldh3*-mediated molecular mechanism in tolerating FSA stress. This novel mechanism alleviated FSA stress by regulating the TCA cycle rates through the modulation of GABA content.

### 3.6. Enhancing Th Resistance to FSA Is Beneficial for Reducing the Occurrence of Plant Diseases

**Figure 6:**
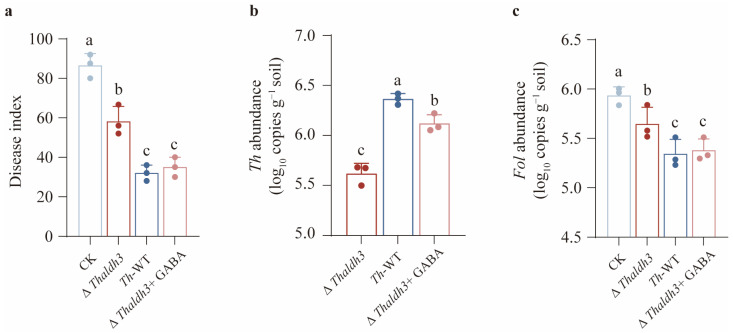
Enhancing *T. harzianum* NJAU4742 resistance to FSA is beneficial for reducing the tomato disease index. The disease index (**a**), *Th* abundance (**b**), and *Fol* abundance (**c**) were measured among different treatments. The data represent the means ± s.d.; *n* = 3 (**a**–**c**). Different letters above error bars indicate a significant group (*p* < 0.05, one-way ANOVA, Duncan’s multiple range tests) (**a**–**c**).

Based on the hypothesis that the biocontrol efficacy of *Th* against *Fol* might correlate with its tolerance to FSA, we conducted a series of pot experiments to validate this hypothesis. Our results demonstrated that a single *Fol* inoculation caused significant disease development in tomato plants, whereas the exogenous application of different *Th* strains alleviated the disease symptoms to varying degrees (*p* < 0.05, Figure 6a). Specifically, the *Th*-WT and ∆*Thaldh3 + GABA* treatments exhibited the strongest disease suppression, with a significant reduction in the disease index by 63% and 59.5%, respectively (*p* < 0.05). In contrast, the ∆*Thaldh3* treatment showed a 32.7% reduction in the disease index. These findings suggest that the efficacy of *Th* against *Fol* is significantly positively correlated with its tolerance capability towards FSA.

In addition, we measured the abundance of *Th* and *Fol* in rhizosphere soil across different treatments. We observed that the abundance of *Th* exhibited a pattern across the three treatments as follows: ∆*Thaldh3* < ∆*Thaldh3 + GABA* < *Th*-WT (Figure 6b). We also found that the highest abundance of *Fol* was observed for the CK treatment, followed by the ∆*Thaldh3* treatment, while no significant differences were detected between the *Th*-WT and ∆*Thaldh3 + GABA* treatments (Figure 6c). A further correlation analysis revealed a significant positive correlation between the abundance of *Fol* and the disease index, while the *Th* abundance was significantly negatively correlated with the disease index (*p* < 0.05, Supplementary Figure S5). These results collectively demonstrate that *Th* could further enhance its abundance in rhizosphere soil by improving its tolerance to FSA, thereby conferring a higher disease resistance to host plants.

**Figure 7:**
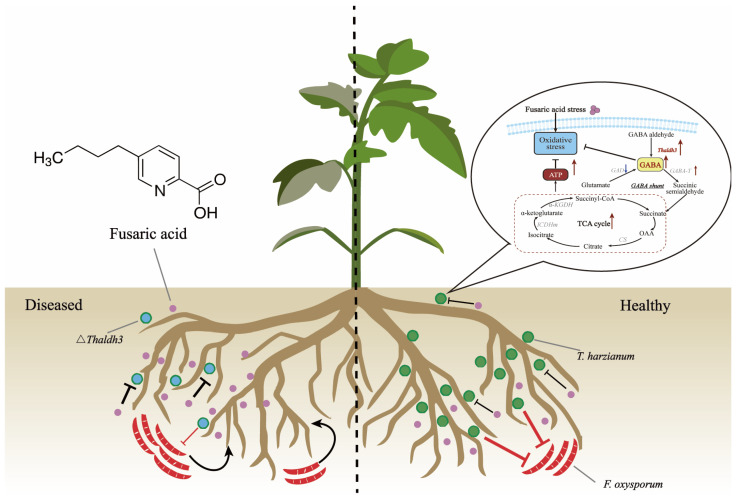
Conceptual diagram of *T. harzianum* NJAU4742 mitigating *Fusarium* wilt disease through enhancing the resistance to fusaric acid (FSA) in the tomato rhizosphere. Under FSA stress, *T. harzianum* showed an increased expression of *Thaldh3*, promoting GABA synthesis. The increased GABA further entered the TCA cycle, resulting in elevated ATP content, enhanced fungal growth, and improved antagonistic activity against pathogens. The red arrows indicate the increase in gene expression levels, enzyme activity or the content of specific substances. The green dots represent wild-type *Trichoderma* (*Th*-WT), while the blue-centered green dots indicate genetically edited *Trichoderma* (∆ *Thaldh3*), and the pink dots represents FSA secreted by *F. oxysporum*.

In conclusion, we propose a working model illustrating how *Th* enhances its antagonistic ability against pathogens by increasing its resistance to FSA in the tomato rhizosphere (Figure 7). Upon exposure to FSA stress resulting from secretions by *Fusarium* species, *Th* enhances the expression of the *Thaldh3* gene, which promotes the biosynthesis of GABA. GABA can subsequently enter the TCA cycle to increase the intracellular ATP content, thereby increasing the *Th* biomass and ultimately enhancing its antagonistic ability against pathogens.

## 4. Discussion

Mycotoxins play multiple complex roles in the interactions between beneficial microbes and pathogens [28]. Mycotoxins are primary regarded as virulence factors employed by pathogens to invade their hosts [8,28,29]. However, with the advancement of our understanding, we have gradually come to realize that mycotoxins can not only serve as "chemical weapons" for pathogens, but can also act as a driving force for the adaptation and evolution of beneficial microbes [17]. Through prolonged ecological competition, many microorganism communities have gradually developed sophisticated mechanisms for metabolizing and adapting to these mycotoxins [30]. For instance, numerous fungi have been reported to detoxify various mycotoxins through diverse pathways or activate defense-related genes to resist or actively transport mycotoxins [28,31,32]. In summary, these interactions mediated by mycotoxins encompass various mechanisms, such as antagonism, competition, and adaptation. Understanding these mechanisms not only sheds light on the intricate interactions within microbial ecosystems, but also establishes a robust theoretical framework for devising more effective biocontrol strategies and integrated disease-management solutions.

In this study, we focused on elucidating the tolerance mechanism of a widely reported fungal biocontrol agent, *Th*, against the mycotoxin FSA secreted by *Fusarium* species. The tolerance of *Trichoderma* to FSA has been previously reported. For example, Marinella reported that FSA strongly inhibited the growth of *Th* ITEM 908, while its UV-C mutant significantly enhanced its tolerance to FSA [17]. Similarly, Vivek tested five different *Trichoderma* strains and found that all of them could tolerate FSA concentrations of up to 500 ppm [33]. He hypothesized that various physiological responses within *Trichoderma* might contribute to their tolerance to FSA. However, these studies did not further elucidate the molecular mechanisms of the *Trichoderma* tolerance to FSA. In our study, we first confirmed that FSA stress induced oxidative stress in *Trichoderma* by measuring the intracellular GSH/ GSSG ratio, consistent with previous reports in both plants [34] and animals [35]. By combining a transcriptomic analysis with functional validation, we further demonstrated that *Th* significantly upregulated a series of antioxidant-related genes under FSA stress, particularly the *Thaldh3* gene. The deletion of *Thaldh3* significantly impaired *Th* growth in the presence of FSA, highlighting its critical role in FSA tolerance. The *Thaldh3* gene is generally associated with the metabolism of toxic aldehydes in various organisms [36], playing a crucial role in mitigating cellular oxidative damage. Our findings also confirmed that the deletion of *aldh3* exacerbates FSA-induced oxidative stress in *Th*, reinforcing its essential role in the fungal defense mechanism against mycotoxin stress.

*Thaldh3* is considered responsible for converting 3-aminopropanal into 3-aminopropionic acid; we thus hypothesized that 3-aminopropionic acid might be a key compound used by *Th* to fight against the oxidative stress caused by FSA. However, our results revealed that 3-aminopropionic acid did not alleviate FSA-induced oxidative stress. Instead, *aldh3* significantly influenced the intracellular GABA content, thereby modulating *Th*’s tolerance to FSA. Studies have shown that members of the *aldh* family exhibit a broad substrate specificity [37,38], catalyzing the oxidation of multiple aldehydes. This also explains why we observed a decrease in the GABA content in ∆*Thaldh*3. Although a complementation strain for Δ*Thaldh3* was not included in this study, the observed phenotypes, such as impaired growth under FSA stress, a reduced antioxidant capacity, and decreased GABA accumulation, were consistently reversed by an exogenous GABA application. This phenotypic rescue supports the functional role of *Thaldh3* in GABA-mediated FSA tolerance. Nevertheless, future studies incorporating complementation lines will be valuable to further confirm the specific contribution of *Thaldh3*.

In this study, we also confirmed that *Thaldh3* enhances the *Th* resistance to FSA by increasing the TCA cycle rate, further contributing to ATP accumulation. GABA produced by *Thaldh3* can directly enter the TCA cycle via the GABA shunt, thereby accelerating energy metabolism [39]. This process not only maintains the physiological activity of *Th*, but also enhances its antioxidant capacity under FSA stress. Our study provides additional evidence supporting the pivotal role of GABA as a signaling molecule in various stress environments [40]. Although our results highlight the central role of GABA metabolism in mediating *Thaldh3*-dependent FSA stress tolerance, microbial responses to chemical stressors are often multifactorial. It is possible that other downstream metabolites or parallel pathway, such as glutathione turnover [41] or redox-sensitive signaling cascades [42], may also contribute to the observed phenotype. Further metabolomic and genetic studies will be required to dissect the broader metabolic networks involved in FSA-induced oxidative stress adaptation.

In the competition between pathogens and beneficial microbes, pathogens often rely on secreting mycotoxins to inhibit the growth of beneficial microbes [29]. This strategy allows them to occupy advantageous ecological niches and subsequently infect plants [29]. Therefore, improving the tolerance of beneficial microbes to these mycotoxins is of great significance for biocontrol efficiency and maintaining plant health. Our pot experiments demonstrated a significant positive correlation between *Th*’s effectiveness in controlling *Fol* and its tolerance to FSA. Enhancing *Trichoderma*’s resistance to FSA facilitates its numerical dominance in the rhizosphere, thereby improving its antagonistic activity against pathogens. Similarly, Marinella found that enhancing the tolerance of *Trichoderma harzianum* ITEM 908 to FSA is beneficial for improving its effectiveness in disease control [17]. Notably, although the *Th*-WT treatment exhibited a higher *Th* abundance than the ∆*Thaldh3* + GABA treatment, no significant differences were observed between these two treatments in terms of the disease index or the *Fol* abundance. A previous study indicated that the application of GABA can reduce the disease index by altering the rhizosphere’s microbial community [43]. Therefore, we hypothesize that *Trichoderma* and GABA acted synergistically to maintain plant health by reshaping the microbial community in the ∆*Thaldh3* + GABA treatment. Future studies should focus on exploring the regulatory effects of *Trichoderma* and GABA on the microbial community structure. In conclusion, our study revealed a novel mechanism by which *Thaldh3* enhanced the biocontrol efficacy of *Trichoderma* through the GABA-mediated mitigation of FSA stress, providing new insights into fungal stress adaptations and biocontrol improvement.

## 5. Conclusions

This study revealed that the regulation of redox metabolism, particularly through GABA metabolism mediated by *Thaldh3*, is a key molecular mechanism underpinning *Trichoderma harzianum*’s enhanced survival and antagonistic capacity against FSA produced by *Fusarium* species. These findings significantly advance our understanding of beneficial microbial defense strategies against mycotoxins and provide a crucial theoretical foundation for enhancing the stability and efficacy of biocontrol agents to promote plant health.

## Data Availability

The original contributions presented in this study are included in the article/Supplementary Material. Further inquiries can be directed to the corresponding author.

## Supplementary Materials

The following supporting information can be downloaded at: https://www.mdpi.com/article/10.3390/jof11070542/s1, Figure S1: Schematic diagram of gene knockout via homologous recombination using hygromycin resistance as a selection marker; Figure S2: Principal coordinate analysis (PCoA) of transcriptiome profile among CK and FSA treatments; Figure S3: Correlation matrix analysis of transcriptomic profiles across strains and their replicates; Figure S4: The relative expression of genes among CK and FSA treatments; Figure S5: Verification of each gene knockout mutant by PCR to verify homologous recombination and whether gene exists; Figure S6: The Pearson correlation between Disease index and *Fol* abundance (a). The Pearson correlation between Disease index and *Th* abundance (b). Table S1: Oligonucleotide primers used in this study; Table S2: Sequencing sequence statistics and quality control; Table S3: The basic information of eight genes involved in the β-alanine biosynthesis pathway; Table S4: Summary of gene knockout efficiency for eight genes involved in the β-alanine biosynthesis pathway.

## References

[1] Munkvold G.P. (2017). Fusarium species and their associated mycotoxins. Mycotoxigenic Fungi Methods Protoc..

[2] Bakker M.G., Acharya J., Moorman T.B., Robertson A.E., Kaspar T.C. (2016). The potential for cereal rye cover crops to host corn seedling pathogens. Phytopathology.

[3] Ma L.-J., Geiser D.M., Proctor R.H., Rooney A.P., O’Donnell K., Trail F., Gardiner D.M., Manners J.M., Kazan K. (2013). Fusarium pathogenomics. Annu. Rev. Microbiol..

[4] Kokkonen M., Ojala L., Parikka P., Jestoi M. (2010). Mycotoxin production of selected Fusarium species at different culture conditions. Int. J. Food Microbiol..

[5] Proctor R.H., Hohn T.M., McCormick S.P. (1995). Reduced virulence of Gibberella zeae caused by disruption of a trichothecene toxin biosynthetic gene. Mol. Plant-Microbe Interact..

[6] Marroquín-Cardona A., Johnson N., Phillips T., Hayes A. (2014). Mycotoxins in a changing global environment–a review. Food Chem. Toxicol..

[7] Wu F., Groopman J.D., Pestka J.J. (2014). Public health impacts of foodborne mycotoxins. Annu. Rev. Food Sci. Technol..

[8] Liu S., Li J., Zhang Y., Liu N., Viljoen A., Mostert D., Zuo C., Hu C., Bi F., Gao H. (2020). Fusaric acid instigates the invasion of banana by *Fusarium oxysporum* f. sp. cubense TR 4. New Phytol..

[9] D’Alton A., Etherton B. (1984). Effects of fusaric acid on tomato root hair membrane potentials and ATP levels. Plant Physiol..

[10] Telles-Pupulin A.R., Diniz S., Bracht A., Ishii-Iwamoto E. (1996). Effects of fusaric acid on respiration in maize root mitochondria. Biol. Plant..

[11] Niehaus E.-M., Diaz-Sanchez V., Von Bargen K., Kleigrewe K., Humpf H.-U., Limón M.C. (2014). Fusarins and fusaric acid in fusaria. Biosynthesis and Molecular Genetics of Fungal Secondary Metabolites.

[12] Gxasheka M., Wang J., Gunya B., Mbanjwa V., Tyasi T.L., Dlamini P., Gao J. (2021). In vitro effect of some commercial fungicides on mycelial growth of Fusarium species causing maize ear rot disease in China. Arch. Phytopathol. Plant Prot..

[13] Chen L.-H., Cui Y.-Q., Yang X.-M., Zhao D.-K., Shen Q.-R. (2012). An antifungal compound from Trichoderma harzianum SQR-T037 effectively controls Fusarium wilt of cucumber in continuously cropped soil. Australas. Plant Pathol..

[14] Xu S., Liu Y.-X., Cernava T., Wang H., Zhou Y., Xia T., Cao S., Berg G., Shen X.-X., Wen Z. (2022). Fusarium fruiting body microbiome member Pantoea agglomerans inhibits fungal pathogenesis by targeting lipid rafts. Nat. Microbiol..

[15] Matsumoto H., Fan X., Wang Y., Kusstatscher P., Duan J., Wu S., Chen S., Qiao K., Wang Y., Ma B. (2021). Bacterial seed endophyte shapes disease resistance in rice. Nat. Plants.

[16] Tao C., Wang Z., Liu S., Lv N., Deng X., Xiong W., Shen Z., Zhang N., Geisen S., Li R. (2023). Additive fungal interactions drive biocontrol of Fusarium wilt disease. New Phytol..

[17] Marzano M., Gallo A., Altomare C. (2013). Improvement of biocontrol efficacy of Trichoderma harzianum vs. *Fusarium oxysporum* f. sp. lycopersici through UV-induced tolerance to fusaric acid. Biol. Control.

[18] Karlovsky P. (2008). Secondary metabolites in soil ecology. Secondary Metabolites in Soil Ecology.

[19] Meca G., Soriano J., Gaspari A., Ritieni A., Moretti A., Mañes J. (2010). Antifungal effects of the bioactive compounds enniatins A, A1, B, B1. Toxicon.

[20] Wang Q., Xu L. (2012). Beauvericin, a bioactive compound produced by fungi: A short review. Molecules.

[21] Woo S.L., Hermosa R., Lorito M., Monte E. (2023). Trichoderma: A multipurpose, plant-beneficial microorganism for eco-sustainable agriculture. Nat. Rev. Microbiol..

[22] Zhang J., Miao Y., Rahimi M.J., Zhu H., Steindorff A., Schiessler S., Cai F., Pang G., Chenthamara K., Xu Y. (2019). Guttation capsules containing hydrogen peroxide: An evolutionarily conserved NADPH oxidase gains a role in wars between related fungi. Environ. Microbiol..

[23] Zhang J., Akcapinar G.B., Atanasova L., Rahimi M.J., Przylucka A., Yang D., Kubicek C.P., Zhang R., Shen Q., Druzhinina I.S. (2016). The neutral metallopeptidase NMP1 of Trichoderma guizhouense is required for mycotrophy and self-defence. Environ. Microbiol..

[24] Tripathi P., Singh P.C., Mishra A., Chauhan P.S., Dwivedi S., Bais R.T., Tripathi R.D. (2013). Trichoderma: A potential bioremediator for environmental clean up. Clean Technol. Environ. Policy.

[25] Shelp B.J., Aghdam M.S., Flaherty E.J. (2021). γ-Aminobutyrate (GABA) regulated plant defense: Mechanisms and opportunities. Plants.

[26] Zhao L., Shu Y., Xiao J., Lin R., Godana E.A., Zhang X., Zhang H. (2022). Transcriptome analysis reveals mechanisms involved in the enhanced antagonistic efficacy of Sporidiobolus pararoseus Y16 treated by γ-aminobutyric acid. Biol. Control.

[27] Ji H.M., Mao H., Li S., Feng T., Zhang Z., Cheng L., Luo S., Borkovich K.A., Ouyang S. (2021). Fol-milR1, a pathogenicity factor of *Fusarium oxysporum*, confers tomato wilt disease resistance by impairing host immune responses. New Phytol..

[28] Venkatesh N., Keller N.P. (2019). Mycotoxins in conversation with bacteria and fungi. Front. Microbiol..

[29] Jin X., Jia H., Ran L., Wu F., Liu J., Schlaeppi K., Dini-Andreote F., Wei Z., Zhou X. (2024). Fusaric acid mediates the assembly of disease-suppressive rhizosphere microbiota via induced shifts in plant root exudates. Nat. Commun..

[30] Ianiri G., Idnurm A., Wright S.A.I., Durán-Patrón R., Mannina L., Ferracane R., Ritieni A., Castoria R. (2013). Searching for genes responsible for patulin degradation in a biocontrol yeast provides insight into the basis for resistance to this mycotoxin. Appl. Environ. Microbiol..

[31] Magan N., Marcon A.G., Samsudin N.I.P., Rodríguez-Sixtos A., Garcia-Cela E., Verheecke-Vaessen C., Medina A. (2020). Biological control agents for mycotoxin control: Are they resilient enough?. How Research Can Stimulate the Development of Commercial Biological Control Against Plant Diseases.

[32] Ianiri G., Idnurm A., Castoria R. (2016). Transcriptomic responses of the basidiomycete yeast Sporobolomyces sp. to the mycotoxin patulin. BMC Genom..

[33] Sharma V., Bhandari P., Singh B., Bhatacharya A., Shanmugam V. (2013). Chitinase expression due to reduction in fusaric acid level in an antagonistic Trichoderma harzianum S17TH. Indian J. Microbiol..

[34] Singh V.K., Upadhyay R.S. (2014). Fusaric acid induced cell death changes in oxidative metabolism of *Solanum lycopersicum* L.. Bot. Stud..

[35] Devnarain N., Tiloke C., Nagiah S., Chuturgoon A.A. (2017). Fusaric acid induces oxidative stress and apoptosis in human cancerous oesophageal SNO cells. Toxicon.

[36] Jackson B., Brocker C., Thompson D.C., Black W., Vasiliou K., Nebert D.W., Vasiliou V. (2011). Update on the aldehyde dehydrogenase gene (ALDH) superfamily. Hum. Genom..

[37] Shortall K., Durack E., Magner E., Soulimane T. (2021). Study of ALDH from Thermus thermophilus—Expression, Purification and Characterisation of the Non-Substrate Specific, Thermophilic Enzyme Displaying Both Dehydrogenase and Esterase Activity. Cells.

[38] Brocker C., Vasiliou M., Carpenter S., Carpenter C., Zhang Y., Wang X., Kotchoni S.O., Wood A.J., Kirch H.-H., Kopečný D. (2013). Aldehyde dehydrogenase (ALDH) superfamily in plants: Gene nomenclature and comparative genomics. Planta.

[39] Michaeli S., Fait A., Lagor K., Nunes-Nesi A., Grillich N., Yellin A., Bar D., Khan M., Fernie A.R., Turano F.J. (2011). A mitochondrial GABA permease connects the GABA shunt and the TCA cycle, and is essential for normal carbon metabolism. Plant J..

[40] Li L., Dou N., Zhang H., Wu C. (2021). The versatile GABA in plants. Plant Signal. Behav..

[41] Deng H., Chen J., Gao R., Liao X., Cai Y. (2016). Adaptive Responses to Oxidative Stress in the Filamentous Fungal Shiraia bambusicola. Molecules.

[42] Keller N.P. (2019). Fungal secondary metabolism: Regulation, function and drug discovery. Nat. Rev. Microbiol..

[43] Wang P., Lopes L.D., Lopez-Guerrero M.G., van Dijk K., Alvarez S., Riethoven J.-J., Schachtman D.P., Xu G. (2022). Natural variation in root exudation of GABA and DIMBOA impacts the maize root endosphere and rhizosphere microbiomes. J. Exp. Bot..

