# Supplementary material for: Thaldh3-Dependent GABA Metabolism Modulates Response of Trichoderma to Fusaric Acid-Induced Oxidative Stress
Source: J Fungi (Basel). 2025 Jul 21;11(7):542. doi: 10.3390/jof11070542 (PMC12300697; doi:10.3390/jof11070542)
Supplement: Supplementary file 1 [file jof-11-00542-s001.zip › jof-3707508-SI.pdf]

Supplementary information for

Thaldh3 enhances the biocontrol efficiency of *Trichoderma* by alleviating fusaric acid-induced oxidative stress through GABA synthesis

Linhua Cao[1, 2†], Xiaoteng Shi[1, 2†], Tuo Li[1, 2], Yang Liu[1, 2], Toukai Wang[1, 2], Bozheng Lin[1, 2], Dongyang Liu[1, 2*], Qirong Shen[1, 2]

Affiliations

1. Key lab of organic-based fertilizers of China and Jiangsu provincial key lab for solid organic waste utilization, Nanjing, China

2. Nanjing Agricultural University, Nanjing 21009 5, Jiangsu, China

[†]These authors contributed equally to this work

***Corresponding author:**

Dongyang Liu, College of Resources & Environmental Sciences, Nanjing Agricultural University, 210095, Nanjing, P. R. China E-mail: liudongyang@njau.edu.cn.

Supplementary Figures

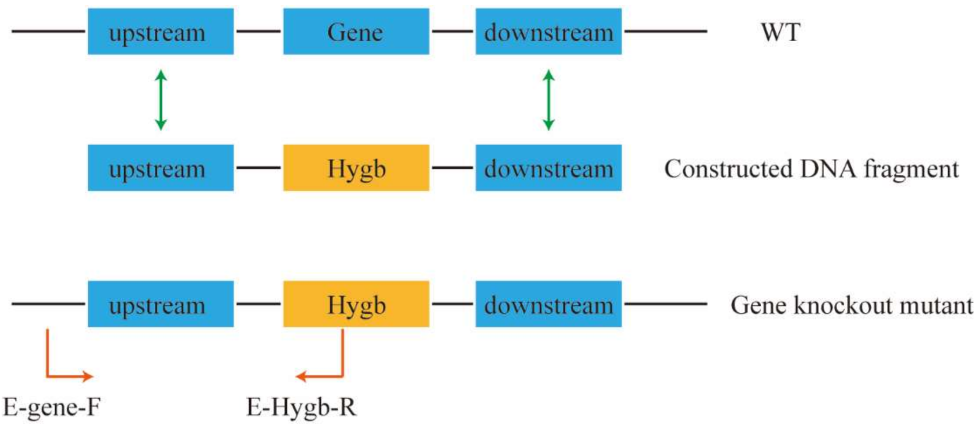

Figure S1. Schematic diagram of gene knockout via homologous recombination using hygromycin resistance as a selection marker. upstream: Gene upstream homologous arm; downstream: Gene downstream homologous arm; Hygb: Hygromycin resistance gene

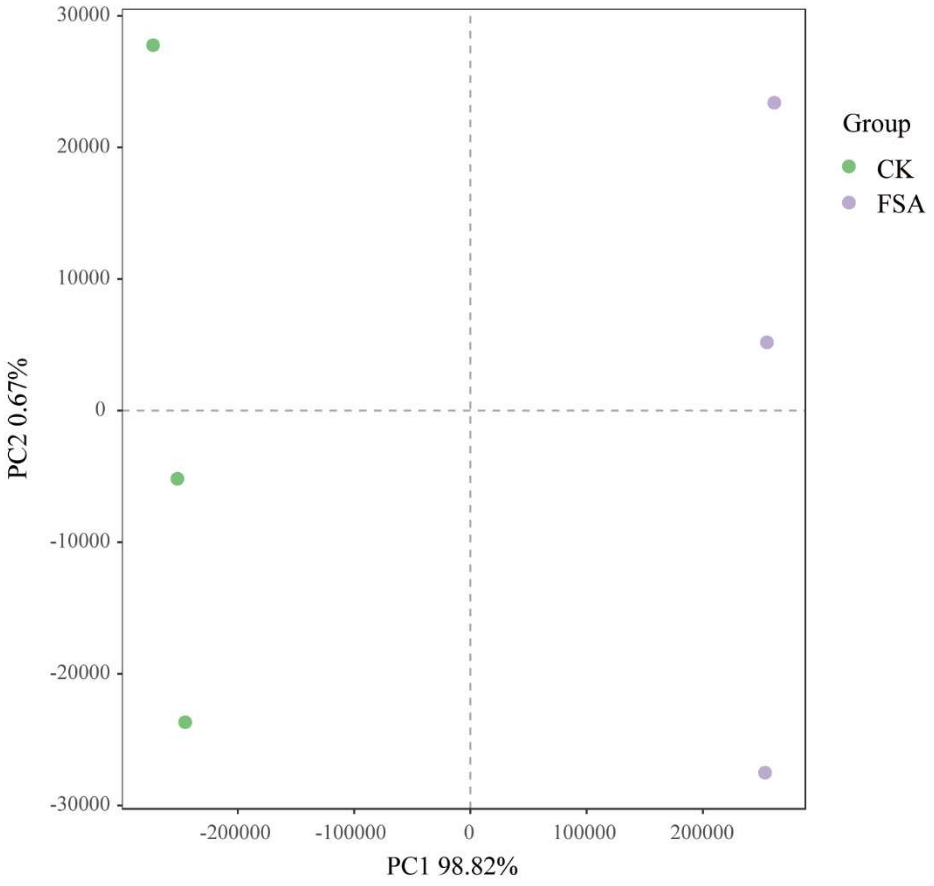

Figure S2. Principal coordinate analysis (PCoA) of transcriptiome profile among CK and FSA treatments. Each treatments contained three replicates.

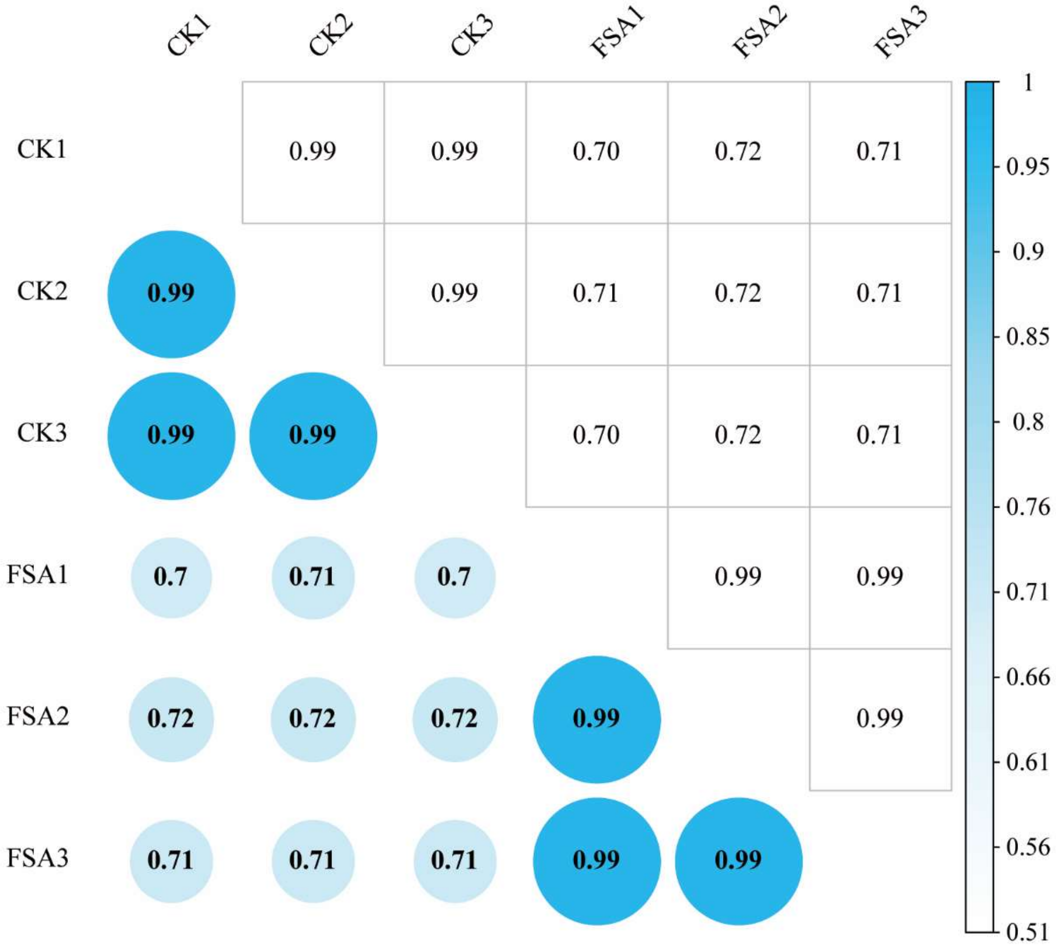

Figure S3. Correlation matrix analysis of transcriptomic profiles across strains and their replicates. Each treatments contained three replicates.

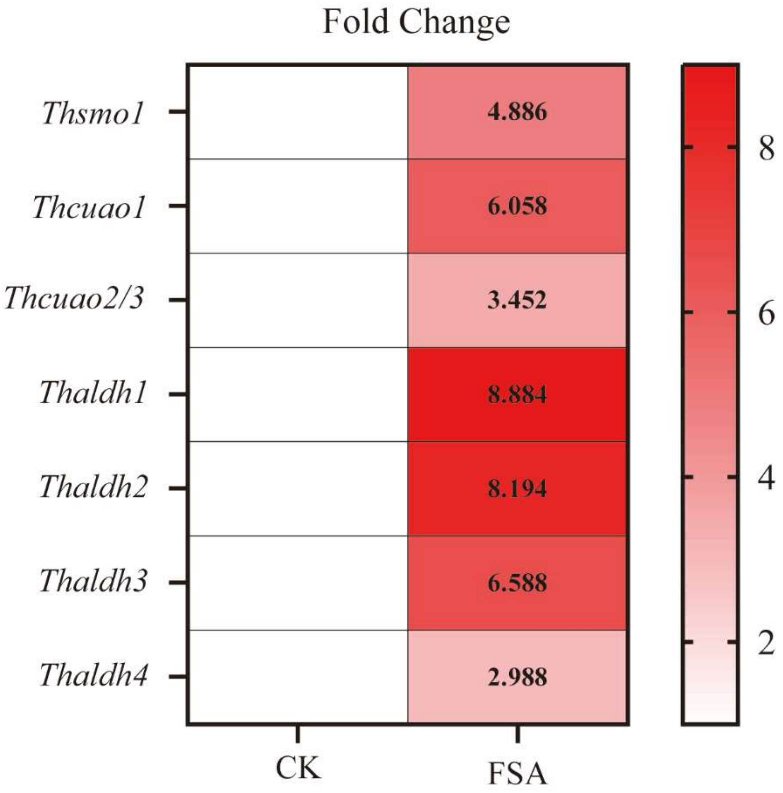

Figure S4. The relative expression of genes among CK and FSA treatments. Each treatments contained three replicates. The gene expression was relative to the CK treatment. The Fold Change are displayed as a heatmap with scale ranges from 1 to 9 (white to red).

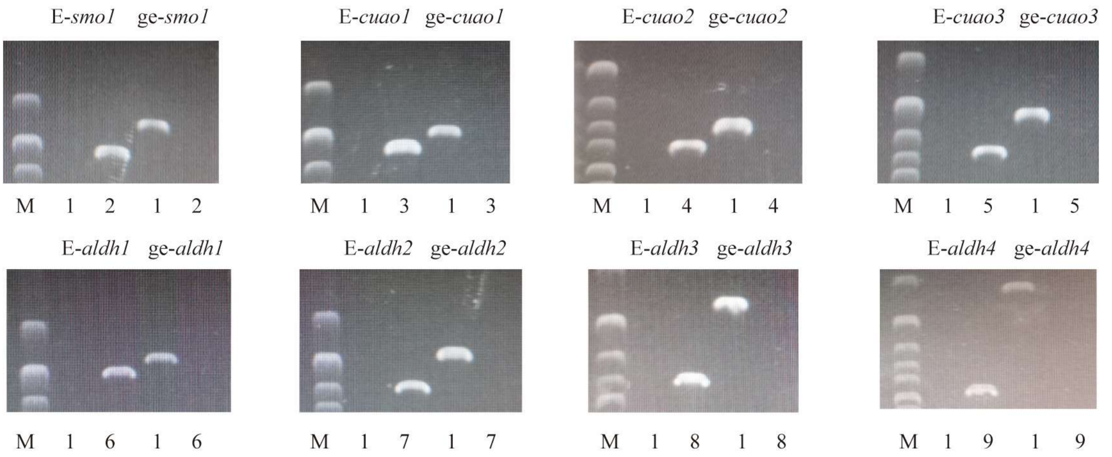

Figure S5. verification of each gene knockout mutant by PCR to verify homologous recombination and whether gene exists.

1. *Th*-WT, 2. Δ*Th-smo1*, 3. Δ*Th-cuao1*, 4. Δ*Th-cuao2*, 5. Δ*Th-cuao3*, 6. Δ*Th-aldh1*, 7. Δ*Th-aldh2*, 8. Δ*Th-aldh3*, 9. Δ*Th-aldh4*

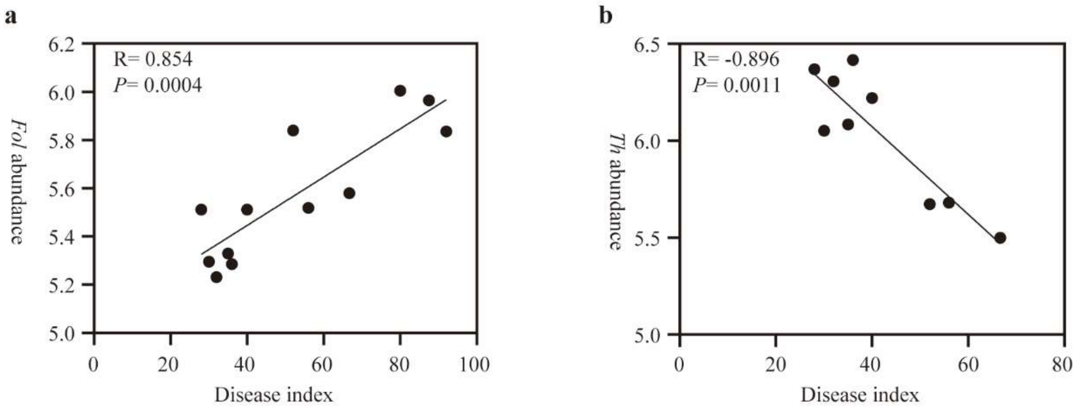

Figure S6. The Pearson correlation between Disease index and *Fol* abundance (a). The Pearson correlation between Disease index and *Th* abundance (b).

Table S1 Oligonucleotide primers used in this study

Primer name	Primer sequence(5'-3')
smo1-up-F	GAGATGACGATGCGACACACAG
smo1-up-R	GGTAGCTCTCATCGATTGGTCGTACCTCTCCCACCTCGTATCTACTCCTC
smo1-do-F	CCATAGTACCACCCCGCCATAAAGCAATGAGTGGTGAATGTGCTTATCTGC
smo1-do-R	CCAATTCAAAAGATCCCCGTGTCG
E-*smo1*-F	GGTCCATGACTGAGGAGAAGAAGG
cuao1-up-F	GCTTCACCAGTTGATTCCGAAGG
cuao1-up-R	GGTAGCTCTCATCGATTGGTCGTACGTGATGCTTTGGACAGACGTTATCG
cuao1-do-F	CCATAGTACCACCCCGCCATAAAGCAGTGAGACACAATTTGCCTCCTAG
cuao1-do-R	GTTTTCGCGCCTTTACCTGTTG
E-*cuao1*-F	GGCTTGTCATTAACCGAGGATCTG
cuao2-up-F	GTCTTCCGACTTCACGGTGATCG
cuao2-up-R	GGTAGCTCTCATCGATTGGTCGTACGAACACTGAGACGAGGAATGGTG
cuao2-do-F	CCATAGTACCACCCCGCCATAAAGCACTAAAAGAGGTACCATTGCCCCC
cuao2-do-R	GGCAGTCAAGTCCTTCCTTCATTC
E-*cuao2*-F	GATGCTATCCTCCGAGGAAAATCGC
cuao3-up-F	GGGACGGGAACTTGGAGATAAGAAG
cuao3-up-R	GTGATTCATCGTGAGTTGCCGTC
cuao3-do-F	GGTAGCTCTCATCGATTGGTCGTACGGAGAGATGATGAGTGGCTATCTGC
cuao3-do-R	CCATAGTACCACCCCGCCATAAAGGCCTCATGTCTTGTTAGTTGCGTTG
E-*cuao3*-F	GAATCACGGCACATGCAGATCG
aldh1-up-F	CCATCTTCTCTCTCATCGGTTGGTG
aldh1-up-R	GGTAGCTCTCATCGATTGGTCGTACCAACCTCCAAATCAGCCAAGGAAAG
aldh1-do-F	CCATAGTACCACCCCGCCATAAAGGAATTGGTTCGGGCTAGGTAGTGAC
aldh1-do-R	CCGTACGATTAGCATCCAGCAAC
E-*aldh1*-F	CAAGTGATGAGCATTCCCATCTGG
aldh2-up-F	CGCATTTCTTTTTCTGGTGCCG
aldh2-up-R	GGTAGCTCTCATCGATTGGTCGTACGAAGATGATGCTGAAGAAGATGAAGC C
aldh2-do-F	CCATAGTACCACCCCGCCATAAAGGGAGGCAGATTAAATCATAATGCATTT CCAC

aldh2-do-R	GGCTCTCGTTGTAACATTTCCGTG
E-*aldh2*-F	CAGGGCTGATGGGTAGATTTGC
aldh3-up-F	CAAGGCTTGATGGTGTGAACGAAC
aldh3-up-R	GGTAGCTCTCATCGATTGGTCGTACGTAAAAAGACGAGAGGGGCAGG
aldh3-do-F	CCATAGTACCACCCCGCCATAAAGCGATTAGTATGAGTACTGGGCAGGG
aldh3-do-R	GCAGCAAGAAAACACAGCCAAG
E-*aldh3*-F	CAACAACTGAGCTCTCGGTGAG
aldh4-up-F	CTGCATCTTTATCGCCTCACGG
aldh4-up-R	GGTAGCTCTCATCGATTGGTCGTACCCCCACGACATATTATCTAGCTGGC
aldh4-do-F	CCATAGTACCACCCCGCCATAAAGGGCGCGATAGAACTGAGAAGATG
aldh4-do-R	CACGGACATCGACTATTGGCTCTC
E-*aldh4*-F	AGTATTTGAACAAGATCAAATcCCCCCA
Hygb-F	GTACGACCAATCGATGAGAGCTACC
Hygb-R	CTTTATGGCGGGGTGGTACTATGG
E-Hygb-R	CCTGGCAAAACGGTATCATCAAC
Q-*Th*-F	GTGGCGTCCTTGGTCATTG
Q-*Th*-R	ACACAGAGCGTAGGCATAGAT
Q-*Fol*-F	GGGAGCCCCAGATATTTTTCA
Q-*Fol*-R	GGATGCTGCCACCTTATCCA
smo1-F	GTTGAATGTCAGAGGCTTGGAGAG
smo1-R	CTTTGTATACGTGCCCATGGCG
cuao1-F	CGCCCGCTAGGATCTATTTTACC
cuao1- R	GCAGCCTTTCTATGGTAAGGCG
cuao2-F	ATGGCAACACACATTCATCCTTTCG
cuao2- R	TTAAGAACCACAGCAAACCTCTACTTTCTTC
cuao3-F	CAAGAGCCGAAAAAAGCAGACCTG
cuao3- R	ATGACCTTGTAGCCCAGGTTCTTTC
aldh1-F	CAACGTTTATACTGTGCCGACGTG
aldh1-R	CAGTACTGTCGCGTGCTGAATC
aldh2-F	CCGAGGTGGATGTTGATATTGCTG
aldh2-R	CTCCTCCTGGGCGATCTTCATATC
aldh3-F	CGACAAGAAGAAGTTTGAGGTCGTC

aldh3-R	GCTGGATGAAGTAGCCCTTCTCAC
aldh4-F	CGAGACAATCGCATCCATCGATC
aldh4-R	CGGCCTTTAGGGTTTCGACAAATC

Table S2 Sequencing sequence statistics and quality control

Sample	Raw Reads	Clean Reads	Valid Bases	Q30%	GC content%
CK-1	51.69	50.86	94.67%	95.07%	53.77%
CK -2	48.89	48.11	94.00%	95.06%	53.77%
CK -3	48.07	47.27	93.86%	95.22%	53.75%
FSA-1	47.29	46.48	94.30%	95.17%	53.69%
FSA-2	48.06	47.29	93.64%	95.30%	53.85%
FSA-3	50.96	50.13	94.17%	95.18%	53.68%

Table S3 The basic information of eight genes involved in the β-alanine biosynthesis pathway

Gene name	NCBI accession	Gene length (BP)	Gene (Introns)
smo-1	OPB45744.1	2008	ATGGCACCAAATAACTTTAAAATCGGTGTGGTTGCAGTGCTTGCGGGATGGATTCCGTCAGTTACTGCCGCACCAGTT<br>AGCAGTAATAACGGGCAAACATGCAGGAAGACATCTGTTGCGATATTGTGAGTATCTTTGAGACTCAATGagttgaatgtca<br>gaggcttggagagtgtcgcttacatgtttgcagaggtgcaggaatggcaggcatcactgctgctgtaagtcttattatcagctatcactttctataaaactaactgttCGTGTAGCAAGCC<br>CTACATAATGCCTCGGTGTCGGACTTTGTCATTATTGAGTACAATGATCGAATTGGTGGACGGGCGTGGCATGGCGAC<br>TTTGGCAAAAAGTCAGATGGGAGCCCGTATGTCATCGAGTATGGGTGCAATTGGGTATGTCTCgttccggaacatgtcttttgttgata<br>agatgagatgctaatgtgaatgctctttagatacaaggtctcggaaatcctggaggccctggtaagtggctcgtgatccatgtctttaccacacaatgagttgctaacaatgcatagagaatcccgtg<br>tatacactggtaagtggtgctgttaagctattcttgaaataaCTTGTATCTGTTTGGAACTTGTCTCGCTAACATTGTATGTAGGCCAAAAAATaC<br>CATCTCGAGAACACCTATTCGGATTATGACTCCATCTTGACATATGATGAAACGGGCTTCACAGAATTTTCTGGTCTC<br>ATCGACGAATATGGGACTGCATACGACAAAGCTGCCGCAAAGGCTGGTCGTTTGCTTGTCCAGAACTTGCAGGATCA<br>GACCATGCGTGCGGGATTATCCACTGCGGGATGGAATCCAAAACATGGCGATATGAAGAGACAGGCCGCTGAGTGG<br>TGGAACTGGggtgagttctcaacatgggtagcaacaatctggttattgcccgagatactaggttgtggtatatgcgaatgctgacaatgtcccaGATTGGGAGGCTGCTTT<br>CCCACCCGAAGAGTCATCCTTCATCTTTGGTGTCGCAGGCAGCAACCTCACCTTTAACCAGTTCAGCGATGCCAACAA<br>CTTTGTCATTGACCCCCGCGGCTACAGTGCCATCATCGACGGCGAAGCTTCAACATTTTTTACCAAGAACGATACCCG<br>CCTGCTCCTTAACACTCGTATCACAAACATCACATACTCCGATCACGGCGTCACAGTGTACAATCACGATGGTAGCTG<br>CGTGTCTGCGGACTACGCCATTTCGACCTTTTCACTAGGCGTGCTTCAGAGCAATGCGATTGGCTTCTCGCCACAGTT<br>GCCGGGGTGGAAGCAAGATGCGATCCAGAATTTCGCCATGGGCACGTATACAAAGGTCTTTTTGCAGTTTAATGAGA<br>CCTTTTGGCCGGAGGATACGCAGTACTTTTTGTATGCTTCGCCTACTACGCGGGGCTATTATCCTGTTTGGCAGTCCCT<br>CTCGACTGAAGGTTTCATGCCGGGCTCCAACATCATTTTCGCAACTGTTATTGGCGATGAGTCTTACCGTATCGAGCA<br>GCAGACGGATGAAGAGACAAAAGCAGAGGCTATGGAAGTCCTCCGTCAAATGTTTCCCAATGTCACTATCCCGGAAC<br>CCATTGCGTTCACGTATCCCCGTTGGGCCTCTGAGCCGTGGTCTTTCGGAAGTTACTCAAATTGGCCAGCAGGCACTT<br>CATTGCTAGCTCACCAGAATCTCCGAGCTAATACTGGACGACTGTGGTTTGCGGGTGAGGCTACCAGTGCCGAGTAC

			TTTGGATTTCTTCATGGAGCTTGGTTTGAAGGTCGTGAGGCAGGTGCACAAGTTGCGGCACTTTTGCAAGGCCGACCA<br>TGTGTGCAGTATGGAAATGATAGGCTTTGCGGAGAAAGGAAACATTATGACCCCTTGATCGGGACGTCGCCCTTGAG<br>TCAGTATACTTTGTTGAACGGATGGGCTGTGAGCAGTCTCTATGAGAGTGGAGATGATTGA
cuao-1	OPB45488	2522	ATGGCATCTGCATCAAGCTGCTGTCATAACGGCGATGACCGTGGTGTCTCCTCTGACCTGCCTGCGCCGGAACAACGC<br>CCGCTAGGATCTATTTTACCAGAAGAGATTCGACAGGTGAATTCAATCGGGACTCTAACAATCCATCCGCACTCGAG<br>TAGGGAATACTGATTTAGAATTAATACAGACATCATCTCTTATAAGAGCGCAGTTCACTCCCGGTACTGCCTTTGCGT<br>TTCGAGTGATATCCCTCGTCGAACCACGAAAGGCGGAGCTGAAGCCGCATCTCGAAGCGCTGCGCCGCGGTCTCGGT<br>CGTCCCGACACACCATTTCCTGCTAGAATCACTTTTGCACACTACTATGTCGCGGGCCCGAAAGATTTCTACCGAGCC<br>TATGTCGATGTGTCGGGAAAATCGGCGAAGCTGATAGTCAATGAGAAGCTCGATGCGAAGAGACATGCAATCTCCGA<br>TCCGGCAGAGATGATGCGCGCCGAGAAAGCATGCCTCAATGATCCGCTCATCGTCAAGCAAATTAGCCAGCTTTCGC<br>TGCCATCGGGTAGCCAAGTCATGATTGATCCGTGGACCTACGCCACAGATGAGAAACTTGATGTGGAAAAGAGAACT<br>CTTATTgtaagatgggctcccttatatgacggagagaggaatacctcggctaacatattcgttttctcaagTGTTACtTTTACCTCAAGGGCAATGCTTCCGAA<br>GTTGATGCAAACCACTACGCCTATCCACTGGAtTTTTACTGCCACTTTGACATGGTCAATCTCAAAGTGGTCAAGCTCT<br>TTCGACTGCCGAAGAAGGATTTTGAGCACGAAGTCGAAATTAAGCCGAACGAAGCGCAGTCTGCAGCAGCTGCTCCG<br>TGGGGAACAGTGTCTCTTGATCGCCAAAACGaGTACCATCCAGATTTAAGATCAGGCCCGAAGAAAATCCCAACTCC<br>TCTGCGCGTTGTGCAGCCAGATGGCCCTTCGTTCCGTATTACGGGTAACGTGGTGGAATGGATGGGATGGAAGTTCC<br>ATGTGGGATTCAACTATGTAAGTCTATGTCACTACGTCTAGCCTGTATGCATGTGTCTTACaggaatcccaaacagcgtgagggcatg<br>actttgcataacattaccctcgatggacgcaatacctttatcgcctagcactctcagagtaagcccttttcccagttttcatACCATTGTCATATTAACATGGAGCAGAAT<br>GTTTGTTCCATATGCCGACCCAAGAGCGCCTTACCATAGAAAGGCTGCCTTCGATCTCGGTAGCAACGGTGCAGGTGT<br>CTGTGCAAACAATCTTGCACTTGGTTGCGACTGTTTGGGCCTGATCAAATACTTTGATGCTCATCTGACAGACATTAA<br>TGGCAACCCTCGGGTGATGAAGAACGTCATCTGCTGCCATGAAGTTGACGATGGCCTGCTATGGAAGCACCTCAACT<br>TCAGAACCCAAAAGGCTGCGCTTGTGCGCTCTCGAACTCTCGTTTTGCAAAGCATCATCACAGTATCGAATTACGAGT<br>ACATTCTTGCATTCATCTTTGATACAGCTGGTGCCATCCATTACGAAGTTCGCGCCACCGGAATTGTATCTACGGTGC<br>CCATTggtttgtggatggtccatgatatgatggctcgagttgatgctaaccgccgtctacaGAACAAGGAGTCAAATCTAGCGCCCATGGAACTGTTGTG<br>GCTGATGGTGCAATGGCGCCATACCACCAGCACTTCTTCAACCTCAGAATTGACCCTGCCATTGATGGCCACGAGAA<br>TTCCGTCGTTGTGGAAGAGACGGTTCCCCTTCCAATCAACGAGGGCAACCCTCAGGGCATAGGATACCACGTGAAGC

			AGGAAGTGATAGAAAAAGAAGGATTTGCTGATATCGACCCTCTGAAAAACCGAGTCTTCAAGATCATCAACCCATCC<br>AGCCGCAACCCCGTCAATGACAAGCCAGTTGCATACGCCGTGGTTCCTTTCAATAGCCAGGTAAGTTCCCCATATTAC<br>GTCCCATAAGTAGATGGAATGCCTTTCACGATATCTAATCACTTTGTTTATGTAGCTCATACTTGCCAACCCCAACTC<br>ATTTCACGCGCGACGATCCGAATTCGCCCTACACAGCATGTGGTTTACCAAGTACCACGACGACGAGCTGTACGCGT<br>CTGGCGAATGGACAAACCAATCTGCGGGAGACGAGGGCATATTGACCTGGATCAAGGGCAGGAATGAAGAGATTGA<br>CAACGGCGATGTTGTTGTATGGCATACCTTCGGCACTACTCACAACCCTCGCGTGGAGGTAAGAtgatctgatagcacttggcgcct<br>tttttcctgtttacactcgtctaattttttgcccttaggattggcccgtcatgcctgttgacaagcttcaggtcacactgaagccggtgaacttcttcccgcgaaacccggctattgacttgcccatgagc<br>actcagaaggataacgattccaagttgtacagccgaatgTAA
cuao-2	OPB36163	2159	ATGGCAACACACATTCATCCTTTCGATCCCCTACGGCCTGAAGAAATCAGCCGGgtatgtgtcctaggcaactttacaatgcaaagcaatacta<br>ataaaaatgatggcagGTCGCAGACATTGTGCGGCCCGCGTTCGCCGGCCAGGGTCTCAATTTCCGTGTAATCACTCTCAAAGA<br>ACCGCCTAAAGCTGAGATGATAGATTTTCTTGAGCGTGAGCATCGCAACGAGCCGGAGAGAAAAAAGCCCTCGCGCT<br>GCGCACGGGTTGAAGTGCTGGTGAAGCCTCAAGCTGGCAAACACCAATTGCATGGACTTTTTGTCAATCTCGATGAA<br>AgCAAGATCATAGCGCAAAAGCATCTTATCGGGAAGCATTCATATATCGATGCGGCATACATGAAGGAGGTAGAAGC<br>CGCCTGTCTAGCTGATAAGCAGGTGCAGGAGGAGATTCGCAAGCTAGACCTCCCGCCAGGATCAACAGCAATTGTTG<br>AGCCTTGGGCATATGCAACAGATGGCGCGAATGATATGAGCGAGCGCATTACtatggtgagaaatccattactacacatttttttgactgaatactg<br>atgcatttttgaaCAAGTGCTGGTTTTATTGCCGTCATTTTGATCATCCAGATGTCAACTATTACGCTTATCCTCTCGACATATG<br>TGCCGAGATATCAGAGCAACTTCAAGTCATTCGAGTTTATCGCTTGCCTGGAACAAAAGATGAAAAGATTAATAATG<br>AAGATCGCCCTTATGATCGCCGCAAAATACATGAAGCGTCATCCAGCGAATATCATCCCGATCTACGACCCAAGCCG<br>CGATCTACCATCAAACCCTACCAGGTGATACAGCCTGACGGCCCCTCGTTTCAAATTCAAGGCAACCTGCTGAACTG<br>GGAAAAATGGAGTTTTCGCGTCGGTTTCAACTATCGAGAGGGCCTGACACTCCATGATATACGGTATGATGGCCGGA<br>GTCTTTTCTACCGTCTCTCCTTGGCAGAAATGTTTGTTCCTTACGGCGACCCACGGGCTCCATACCCACGAAAGGCTG<br>CTTTTGACCTCGGCAACGATGGTGCCGGCATAAACGCAAATAACTTGCAGCTTGGCTGTGACTGTCTAGGTACCATCA<br>AATATTTTGATGCTTGGCATAATACTCAATCTGGAGAACCTATGAAGCTGCCCAACGTTGTTTGCTGTCACGAACAAG<br>ATGACGGAATCCTATGGAAGCACACCAACTTTCGTACCGGTAATGCGGTGGTCACTCGCTCACGCATCCTCGTGTTGC<br>AGACAATCATCACAGTGAGCAATTATGAATACATTTTTGCTTTCCAATTCGCTCAGGACGCATCCATCTTATATGAAG<br>TTCGGGCAACTGGCATTCTGTCCACTGTACCACATCACCTAGACCAGAAGGACAAGGTTCCATATGGCACAGTAGTC

			GCACCCGGCGTGTTGGCGCCTTATCATCAGCACCTATTCAGTCTTCGGATTGACCCGGCTGTCGATGGTCATAAAAAT<br>ACATTGTCAATTGAAGAATCTCACCCTATGCCAATTAATGATCCAGCAATTGACAATCCCTTTGGAGTGGGTTATCAC<br>ACTGTGAATCAGTACGTGGAAAAAGAGGGCGGTTTCGATCTGGATATAAGCAAGGGTCGCGTCTTCAAGTTTATCaA<br>CGAAAACAAGACCAACCCAATCAcGGGGACCCCTGTCGGCTTCAAGTTGCTTCCACAGCCAAGTCAGATGCTACtTTC<br>GCATGCAGACTCATATCATGCAAAAAGATCCGAATTTGGTCAACATGCTGTCTGGGTCACTCATTAtGAAGACGACGA<br>CCATTTCCCATCAGGTAGATATACGATGCAATCTTCTGGCGGTGATGGTATTGCTTCAAGTATCCAGAAGCGAAATGA<br>CCTAGGTACCTCTAAATCCATTAGGAACGCAGATATCGTCGTTTGGCATACGTTTGGATCAACACACAATCCTCGAAT<br>CGAAGATTGGCCTGTCATGCCAAGTGAGAAAATGACGGTTGGTCTGAAGCCAGTCAACTTTTTCACAGGCAACCCGG<br>GCTTGGACGTTCCTGTGTCAACACAAGAAAAGAATAAGAGCGTTTTATACACAGAGAATTCAGAGAAGAAAGTAGA<br>GGTTTGCTGTGGTTCTTAA
cuao-3	OPB45312	2382	ATGGCCGTCACCGCAGCTCCGAGGCCGCATCCGCTGGCTCCACTCTCCGAGGCCGAGCACATCAAGGCCCGGGACGC<br>CGTTGCTAAGCTTCACGGCGCCTCCGAGTCCATCTTCTTCAGAGCAATCCACGTCCAAGAGCCGAAAAAAGCAGACC<br>TGCAGCCTTTCCTCGAGGCCGAGCACACGGGCACTCTGACAGAAGAGACCAAGCGGCCGCCCAGAGAGGCCATTGTC<br>GAGCATGATGTTATTCGGGCTGATCGCTCCGAGTACATCAACGCCATTGTCAACTTAGATACCGGCGAAGTCAAGAC<br>AGTAGCCGCTCCGGTGCCTCACCAGCCATATGTCACTCCGtaagtctccccttagaacatgatcgagaaaaagagataggagctctaacacaatatagtG<br>GCGAGTTTGACCACTTTAATGACGCTTGCGTCAACTCGGATCTGTTCAAAAAGGCCATGTCCGAGTTCACCCTTCCAG<br>AAGGCTTTGAAGTCTGCGTCGAGCCATGGCCTTATGGACCCCCAAATGCCGATGAGGAAAACATTCCACGACAGATG<br>CAGGGACTAGTCTACGCAAAGGACACACGCAACAAGAATCCCGACTCCAACCACTACGGCTACCCAATTCCCATCAT<br>CCCCGTAATGGATTGGCATACCAAGAAGCTCGTCAGAGTCGAGAGAATCGCAACTGGCGGCATTGGCGATGAACTAG<br>AGGCCAAGGTACAGAGCGAGGAGCCTGTCAAGCTGTTCGAAAACCACAAGGGCTGCGAGTACGTCCCTGAGCTGCT<br>GGACTATCCTCTGAGAAAGGACCTGAAGCCTATCAACATCACACAGCCCGAGGGCGCTTCCTTTACTATTCACGACG<br>ATGGTCTGATTGAATGGCAAAAGTGGCGATTCCGCCTCGGATTCACTCCCCGTGAAGGTGCGGTCTTGCACGATCTTC<br>ACTACGACAACCGCTCCGTCATGTACCGCCTCAGCTTCAGTGAGATGACGGTCCCTTACGCCGACCCGCGACCTCCCT<br>TCCACCGCAAACAGGCATTTGACTTTGGTGACGGTGGCATGGGACGAGCCGCGAACAACCTGGAGCTGGGATGTGAC<br>TGTCTGGGCGCGATTCACTATATCGACGTGGTCAACACCGAGCCCGATGGCTCACCATCTCCTGGAAAGGCTGTGGT<br>GTGTCTGCACGAACAGGACAATGGCATCCTGTGGAAGCACACCAACTACCGCACCGGCCGAGCCGTTGTGACGCGCA

			ACCGCGAGTTTGTGGTGCAGTTCATTTGCACTCTGGCCAACTACGAGTATGTCCTGTGCTATAAGCTGGACCTTGCCG<br>GTGCCATTACCTTTGAGACCCGCGCTACCGGCATTGTCAGCGTCACTGGTATCGATGAAGGCAAGGTCAGTGCCTAC<br>GGCAACGTCATGACGCCGGGTGTCTTGGCCCAAAACCACCAGCACGTCTTCGCCGTGCGCATCGACCCTGCCATTGA<br>CTCATACGACGGTGCCGATTCGCAGGTCATCGTTGAGGAgTCTCACGGGCAAAAGATTGACCCCAAGACCAACCCCT<br>ATGGCAACTTTTACAAGATTCAGCGCGAAAAGGTCGAGAAGGCTACCTGGGTCGATGCCGAGCCACGCCTCAACCGA<br>CTGATCAAGCTGGAAAACGCCAACAAGAAGAACCCCATCTCAGGAAAGAACCTGGGCTACAAGGTCATGGCACCAG<br>TGACTCAGATGCTTCTGTGCGACCCCGAAGGCTTGGCTGCCCAGCGAGCGCAGTTTGCTCAGCACAATGCCTGGGTG<br>ACTGGTTACCGAGATGGCGAGCTGTGGGCTGCAGGAGAGTTTACGAACCAGAGCACAAAGGAGATTGGTGGTGTTG<br>CCGACATGGTCAAGCGAGGAGACTGGTTCTCAGATCGAGAGGCTAATGGTGCAACGAATGGCGATGCGGCGGGCAA<br>GAGGAGCAGCCCTGTTGTGTGGAGCGTGTTTGGCTTGACACACAATCCCCGTGTTGAGGACTGGCCGGTCATgtaagtcaa<br>ccattgtgttctttgtaaatctccaacatgttgctaacaattactttccatctagGCCAGTTGAGACTTtCCAGATCCACATCAAGCCTACCGACTTCTTCA<br>CCGCCAACCCTGCAATGGATGTTCCCTCTACCAGGAACGACGCCAGTGTTCTGCTCGGAGGATCTTGCTGCACCACAT<br>CAAGCGACAAGAATAAGTCTGGCGTGCAGAACAACCCGCTGACGCACCAGCAGGGAAGTGGCGCCACCATTGACGC<br>CAAGACGGCGGGAGCAAATGTTGACGAGAAGCTGAGCAAGAGGCTAAGCAAGACATTTAACGGATTGTTTGGATCC<br>AAGAAGGAGGAGGTGAACTAA
aldh-1	OPB40776.1	1603	ATGGTTTCCACTTCTATTGAAACGCGGCTGTTCATAAACGGCAAGGTATGAAGTGGCTGCGCAATGTCCTTCTTCTCT<br>AACTCACATCCCACAGTTCCGGCCGTCCTCTAACAATGAGACGTTTGATCTTCTTTCAGCAAAATCTGGTTCATTActag<br>caaaaggtaagatcctctgatcAACGTTTATACTGTGCCGACGTGGGTTCTGACACGACAATGAAACAGTATATGAAGCGACCAAG<br>GAGGATGTAGACGACGCTGTAGCGGCAGCCAGAGCCGCCTTCCCAGCGTGGGCCAGTCTTTCACCGTACGAAAGAGG<br>CAGATATCTCGTGCGGCTCGCCGACCTCATCGTGGAGGCTAATGCAGATCTAGCGCATCTGGAAGCTGAGTCCATGG<br>GTAGACCTGTATCAACGTACTTTGATGCGACCGTGGGTGCCAAGTATTTCCGCTACTTCTCTGAGGCAGCATATCCTC<br>AAGGAGGTTCAAGCCTGAATACTCCGGGATTTGTGAACATTACCTTGAAGCAGCCGGTAGGTGTTGTTGCGGCGATT<br>ATTCCTTGGAATGCACCTCTCGTCTTCTTTTGTAAGAAGCTGGCACCAGCATTAGCCGCCGGCAATGCTGTCGTTCTC<br>AAGAGTAGTGAGAAAGCGCCGTTGACGGTCagttctctttgatacccttcacatctgctccccattctaaaacttccatagtctCTGTACGTCGCTATGCT<br>TGCTCAAAAAGCTGGCTTCCCCCCTGGCGTCCTCAACGTTCTCTCTGGCCATGGCCCCGTCTCGGGCGCAGCGCTCGC<br>TTCGCATATGGACGTCCGGGCCTTGACATTCACTGGCTCCAACCGCACGGGAAAATTAATCGCCAAGATGGCCGCCG

			ATTCCAACATGAAGAACTTGATCTTTGAACTCGGGGGCAAGTCGCCCGCCATCATTTTCGACGACGCCGATATTGATG<br>CCGCGGTACGAGAGACCCAATTCAGCATCCAGCTCATCAGCGGACAGACCTGCATGGCAAATTCGCGCATCTATGTG<br>CAACACTCTATCGCTGAATCCTTTGTCGAGAAGTTTAAGGCTGCCTTCACGTCTGCTCGACTGGGTGATCCGACCGAC<br>CCCCAAGTCAACCACGGGCCGCAGGCGGATAAGATTCAGCACGCGACAGTACTGCGGTATATTGAACTGGGAAAGA<br>AGACGGGCAAGTTGATAACCGACGAGCAGTCTGCTTCGGGCTTATACATCAACCCCACGATATTCAGAGACGTCCCT<br>GAGGATGCTCAGATCATGAAGGAGGAGATCTTTGGGCCAGTGGTGATCATAAACACATTCGACACAGAAGCGGAAG<br>CAGTGGCAAACGCGAACAATACCGAGCACGGTCTGTACGCATCGGTGTATACAAAAGATCTGGATCGCGCGATGAG<br>GGTTGCCTCGAGGCTGGAGGCGGGAACAGTAGGGGTTAACTGCACAAGTCCGACGAAGGGAGACGACATGCCATTT<br>GGTGGGCAAAAGGGGAGTGGAGTGCAAAGGGAGAGTTATATCCATAGTATTGAGACATTTATGGAGACGAAGTCAG<br>TTTTGATCAAGGTGGCAGGGCTATGA
aldh-2	OPB45929.1	1661	ATGGCACTTTTTACAACTATCAAGACTCCCACTGCAACATATGAGCAGCCCCTTGGCCTGTGAGTATCAAGATTCTCC<br>GATTCTACCCACTGTGATCATTTATTCTAATGTTTGCTGTCAGTTTCATCAACAATGAGTTCGTGAAGGGAACTGAAG<br>GCAAATTAATTGAAACTTACAACCCTGCGGATGAGAAACTTATTACCTCTGTCCATGAAGCCACCGAGGTGGATGTT<br>GATATTGCTGTTGCTGCGGCACGCAAGGCCCTCGAAACCACATGGCATCAAATCACCCCATCTGAGCGCGGTCATCT<br>GCTCATGAAGTTGGCGGATCTTTTCGAACGCGATTTCGAAACTCTTGCGGCTATTGAGTCTCTTGACAACGGCAAGGC<br>ACTGAGCCTGGCGAGGATCGATGTTGCCGGCGCTGCTGGTTGCCTCCGATACTACGGTGGCTGGGCAGACAAGATTC<br>ACGGTCAAACAATCGAAACAGATCCCAATTCCCTCAACTACACTCGTCATGAGCCTATTGGCGTCTGCGGTCAGATC<br>ATCCCATGGAACTTCCCTCTTCTTATGTGGGCTTGGAAGATCGGCCCCGCGGTTGCGGCTGGTAACACTGTCGTTCTC<br>AAAACCGCTGAACAAACTCCTCTTTCTGGTCTCTACGCATCCGCATTGGCAAAAGAGGCCGGATTCCCCGCTGGTGTA<br>ATTAACACTATTTCCGGCTACGGTCGTGTTGCAGGATCCGCAATCTCCAGTCACATGGACATTGACAAAGTGGCCTTC<br>ACCGGCTCTACCCCCGTCGGACGAACTATCCTCCAAGCTGCAGCGAAGAGCAACCTCAAGAAGGTGACTTTGGAACT<br>CGGCGGTAAATCACCAAACATTATTTTCAACGATGCCGACTTAGACAATGCAATCAAATGGGCCAACTTCGGTATCT<br>ACTACACGAGCGGACAGATCTGCTGTGCAGGCTCTCGTATCCTCGTGCAGGAAGGCATCTATGACGAATTTGTTAAG<br>CGATTCAAGGAGCGAGCGCAACAGAACAAGCTTGGTAATCCTTTTGACGAGCAGACTTTCCAGGGGCCTCAGGTCTC<br>GCAGCTACAATTCGACCGGATTATGGGATACATTGAGGCCGGTAAGCAGGGTGGTGCGACTCTTGCTCTGGGAGGTG<br>GACGATATGGTGACAAAGGTTTCTTCATTGAACCTACTGTCTTTACCGACGTGACGCCGGATATGAAGATCGCCCAG

			GAGGAGATTTTCGGTCCTGTTGCAGCCATCCAGAAGTTCAAGGACGAGGATGAGGCTATCAAGATTGGAAACAACAG<br>CAACTATGGTAAGTAGAGTCTACTGCTATCATATGGGGCATTGTACACTAACGTCAAACATAGGTCTTGCAGCTGccatc<br>catactagcaatgtcaacactgcaatccgcgtctcaaacgctctacaggcagggtatagtcaatactcttcttcgttgtattgaacctagctaacgttattcacagtACTGTCTGGATCAA<br>CCAATACAATATGCTTTCCTACCAGACTCCATTTGGTGGATACAAGGAGTCGGGCATTGGCCGTGAGCTTGGTTCCTA<br>TGCCCTTGAGAATTACACCCAGGTTAAGTCTGTTCGGTATTCTTTGGTTAATGCTCCATTtGCTTAA
aldh-3	OPB39805.1	1615	ATGGCTCCTTTGACCGTTGAGCTCAACACTCCCATCACGGGCCCCTACTCACAACCCATTGGCCTGTAAGTCCTTGTC<br>CCTCGGTTCACTCCTCTGCTTGATATCAACTAACACTTGGCTTTAAGGTTCATTAACAACGAGTGGGTCGAGGGTGTC<br>GACAAGAAGAAGTTTGAGGTCGTCAACCCCGCCACCGAGGAGGTCATCACCTCTGTCTGCGAAGGTACCGAGAAGG<br>ATGTCGACCTGGCCGTTGCCGCTGCCCGCAAGGCTTTTAACACAACATGGAGAAAGATTGCTCCCGGCGAGCGTGGC<br>CGCCTGATGCTCAAGCTCGCTGATCTCGCCGAGAAGAACCTCGATCTCCTCGCCGCTGTCGAGTCTTTGGATAACGGC<br>AAGTCCATCACCATGGCCCGAGGTGATGTCGGCGCTGTTGTTGGATGCATCCGCTACTACGGTGGATGGGCCGACAA<br>GATTGAGGGAAAGACCCtTGACATTGCCCCCGACATGTTCAACTACACTCGCCAcgagcctgtaagtaacaagagaagccaatcgtctcaga<br>acctgattgctaactacagtcctTGTACAGCTTGGTGTTTGCGGTCAGATCATTCCCTGGAACTTTCCCCTTCTCATGCTTGCATGGA<br>AGATTGGCCCTGCCCTGGCCACTGGTAACACCATCGTCATGAAGACCGCTGAGCAGACCCCTCTGTCAGCCCTCGTCT<br>TCGCCAACCTCGTCAAGGAGGCTGGTTTcCCCCCCgGGGtTTTCAACCTGATCTCTGGTTTCGGCAAGACTGCTGGTGC<br>CGCCATCTCTTCCCACATGGACATTGACAAGGTCGCCTTCACCGGCTCAACCGTCGTTGGCCGCCAAATCATGAAGGC<br>TGCCGCCGCCTCCAACCTGAAGAAGGTCACCCTTGAGCTGGGTGGCAAGTCCCCCAACATTGTCTTCAACGATGCCG<br>ACATTGAGCAGGCCATCTCATGGGTCAACTTCGGCATCTACTTCAACCACGGCCAGACCTGCTGCGCTGGTACCCGTA<br>TCTTTGTCCAGGAGGGCATCTACGACAAGTTCCTCGAGGCCTTCAAGAAGCGCGCTCTGGCCAACAAGGTCGGCGAC<br>CCATTCCACCACGAGACCTTCCAGGGACCCCAGGTCAGCCAGCTGCAGTTCGACCGCATCATGGGCTACATCCAGTC<br>CGGAAAGGAGGAGGGCGCCACGGTCGAGATTGGTGGTGAGCGTCACGGTGAGAAGGGCTACTTCATCCAGCCCACC<br>GTCTTCAGCAACGTCACTGCCGACATGAAGATTATGCGCGAGGAGATTTTCGGCCCCGTCGCCGCCATTGCCAAGTTC<br>AAGGATGAGGAGGAGGTCATCCAGATGGGCAACGACTCCAACTACGGTCTTGCCGCTGCCGTCCACACCAGAGACCT<br>CAACACCGCCATCCGTGTCTCCAATGAGATCCAGGCCGGCACCGTCTGGGTCAACTGCTACAACCTGCTGCACCACC<br>AGATGCCCTTCGGTGGCTACAAGGAGTCTGGTATCGGCCGTGAGCTTGGTGAGGCCGCGCTGGCCAACTACACTCAG<br>AACAAGTCTGTTGCCATCCGTCTGGGAGGCGCTCTGTTCTAA

aldh-4	OPB46540.1	1640	ATGGCTCCTCCCTCGGTCGACCTCACTGCGCCAAATGGCGTCAAATGGAGCCAGCCCACTGGACTGTTTATTAACAATGAGTTTGTTGCATCGTCCAACGGCGAGACAATCGCATCCATCGATCCAGCGtaaggaaacaaatgacagcagctgaggcttctatcaagctgaatttcactaatgcgatcaaatagcACCGAAGAAGTCATTGTCAACGTTCAGGCGGCCAGTGCCGAGGATGTGGACAAAGCCGTGAAAGCGGCAAAGGCAGCATTAAAAGATGCTTCCTGGAAGCAGCTGTCAGCTTCTGATAGAGGCCACCTGATGACACGTCTCGCCGACTTGATTGAGGCAAAGAAAGAGCTCTTTGCGACAATTGAGGCATGGGATAATggtgagagatttcattgtctcatcgctctgttgctggcaagtaattgacacgtgcggttcgtttaGGTAAAACATATCAAGAAGCACTGGATGTTGATCTTGTCGAAGCTGTGGCTGTGATACGGTACTACGCCGGCTGGGCCGACAAACAGTATGGCCAGACGATAAGCACAACACATCAGAAATTTGCATACACTCTCCGCCAGCCCATCGGAGTCATTGGACAGATCATTCCGTGGAATTACCCCCTTTCAATGGCTACTTGGAAGTTGGGACCTGCACTTGCTTGCGGTAACACGGTAGTACTCAAAGCCGCAGAGCAGACACCACTAAGTATCCTTGTGCTTGGAGAGCTTATCAAGGAGGCTGGATTCCCGCCTGGAGTAGTCAACTTCGTCAATGGGCTCGGCAAGGATGCCGGATCTGCCTTGGTCAACCATCCACTGGTAGACAAAATTGCCTTTACAGGGTCAACGGCGACGGCATCAAGTATCATGGCGGCCGCATCAAAGACTTTAAAGAACATCACCCTCGAAACAGGAGGCAAATCGCCGCTTGTTGTATTCAAGGATGCCGATATGGATCAGGCCGTCAAGTGGTCTCATTTAGGTATCATGTCTAACCAAGGCCAAATTTGCACTGCTACATCGCGAATTTTAGTCCAGGATGAAGTATACGACGACTTTCTAAAAGGATTTGTCGAAACCCTAAAGGCCGTCAGCAAAGTGGGCAGCCAGTGGGAAAAGGATACATACCAGGGTCCCCAGGTATCCAAAGCGCAATATGATAGAGTGCTGGAGTACATTGACATCGGTAAAAAAGAGGGCGCCAAGGTTGCTGCCGGAGGTCACCCTCTTGACATTGAAGGCAAGGGTAAGGGTTTCTTCGTCGCTCCAACGGTGTTTACCGACGTCACCCCTTCAATGAGAGTCTACCGAGAGGAGATTTTTGGACCTGTCGTTGTTATCCTGCGATTCAAGACGGAGGACGAAGCACTGGAGCTGGCAAATAACACAACATATGGCTTGGGGGCCGCCGTCTTCACGACAGACCTGGAAAGAGCTCACCGCATGGCCGCTGAGATTGAATCGGGAATGGTCTGGATTAATAGCAGCCAAGACTGTGATCCGCGCGTGCCATTCGGAGGCGTTAAGCAGAGTGGTATCGGCCGAGAACTCGGAGAGGCGGGTCTTGAGGCTTATTCTCAGATTAAGGCTGTACATGTCAACATGGGAAATCGGTTGTAA

Table S4. Summary of gene knockout efficiency for eight genes involved in the β-alanine biosynthesis pathway

Gene name	Total Transformants Screened	Confirmed Knockouts (Positive Clones)	Knockout Efficiency (%)
smo-1	24	6	25
cuao-1	20	5	25
cuao-2	18	4	22.2
cuao-3	22	5	22.7
aldh-1	21	4	19.0
aldh-2	17	3	17.6
aldh-3	19	3	15.8
aldh-4	23	4	17.4
